# Maternal Intellectual and Developmental Disabilities and Infant Outcomes

**DOI:** 10.1001/jamanetworkopen.2026.15005

**Published:** 2026-05-27

**Authors:** Catherine Psaras, Rita H. Ryu, Rebecca Baer, Kristin Palmsten, Lori Kroh, Jeanne Townsend, Jaime Barea, Jerasimos Ballas, Gretchen Bandoli

**Affiliations:** 1 Department of Pediatrics, University of California, San Diego, La Jolla; 2 Herbert Wertheim School of Public Health and Longevity Science, University of California, San Diego, La Jolla; 3 Pregnancy and Child Health Research Center, HealthPartners Institute, Minneapolis, Minnesota; 4 Research on Autism and Development Lab, School of Medicine, University of California, San Diego, La Jolla; 5 Clinical Services Department, San Diego Regional Center, San Diego, California; 6 Department of Obstetrics, Gynecology, and Reproductive Sciences, University of California, San Diego, La Jolla

## Abstract

**Importance:**

Intellectual and developmental disabilities (IDDs) include cognitive and adaptive deficits beginning before ages 18 to 22 years. People with IDDs experience disparities in pregnancy and infant outcomes, yet little is known about how risks differ across IDD subtypes or the extent to which associated maternal health conditions contribute to these disparities.

**Objectives:**

To evaluate the associations between maternal IDD subtypes and adverse infant outcomes and to estimate how much these associations may be explained by modifiable maternal health conditions.

**Design, Setting, and Participants:**

This cohort study analyzed births in California from January 1, 2007, to December 31, 2021, using data from the Study of Outcomes in Mothers and Infants, which links vital statistics on births and infant deaths with maternal and infant hospital inpatient and emergency department records. All 6 435 742 singleton births with gestation between 22 and 44 weeks were included. Statistical analyses were completed on February 19, 2026.

**Exposures:**

Maternal IDD diagnoses, including autism spectrum disorder, cerebral palsy, intellectual disability, chromosomal differences, and other IDDs (eg, fetal alcohol syndrome, tuberous sclerosis, and congenital malformations), were identified from hospital records. Potential mediators included prenatal care utilization, prenatal tobacco use, preexisting chronic and neuropsychiatric conditions, and body mass index.

**Main Outcomes and Measures:**

Outcomes included neonatal intensive care unit (NICU) admission, small-for-gestational-age (SGA) birth, preterm birth (PTB) at less than 37 weeks, and very PTB at less than 32 weeks.

**Results:**

Of 6 435 742 singleton births, 4492 were to mothers with IDD diagnoses (mean [SD] maternal age at birth: IDD cohort, 29 [7] years; overall cohort, 30 [6] years). Infants born to mothers with IDD had higher risks of NICU admission (14% [628 of 4492] vs 5% [327 345 of 6 426 048]; adjusted risk ratio [ARR], 2.76 [95% CI, 2.56-2.98]), SGA birth (14% [614 of 4492] vs 9% [569 146 of 6 426 048]; ARR, 1.56 [95% CI, 1.44-1.68]), PTB (16% [717 of 4492] vs 7% [452 177 of 6 426 048]; ARR, 2.34 [95% CI, 2.18-2.51]), and very PTB (3% [122 of 4492] vs 1% [62 290 of 6 426 048]; ARR, 3.20 [95% CI, 2.66-3.86]) compared with infants born to mothers without IDD. Risks were highest for maternal diagnoses of chromosomal differences, other IDDs, and intellectual disability. Preexisting hypertension, epilepsy, and mental health conditions were associated with a substantial portion of the risk, particularly for NICU admission.

**Conclusions and Relevance:**

In this cohort study of births in California, pregnant people with IDD diagnoses had substantially higher risks of adverse infant outcomes. Preexisting hypertension and neuropsychiatric conditions were key variables and may represent modifiable targets for preconception and prenatal intervention.

## Key Points

**Question:**

How do birth and infant outcomes differ between women with and women without intellectual and developmental disabilities (IDDs)?

**Findings:**

In a cohort study comprising 6.4 million California births (2007-2021), infants of mothers with IDDs had higher risks of neonatal intensive care unit admission, preterm birth, and small-for-gestational-age birth; risks were highest for maternal chromosomal differences or intellectual disability. Preexisting hypertension, epilepsy, and mental health conditions were associated with a substantial portion of these risks.

**Meaning:**

This study suggests that maternal IDDs are associated with adverse infant outcomes and that managing chronic and neuropsychiatric conditions may reduce these disparities.

## Teaser

This cohort study used data on more than 6.4 million births to evaluate the associations between maternal intellectual and developmental disability subtypes and adverse infant outcomes and to estimate how much of these associations may be explained by modifiable maternal health conditions.

## Introduction

Intellectual and developmental disabilities (IDDs), including autism spectrum disorders (ASD), cerebral palsy (CP), chromosomal differences, and intellectual disability (ID), affect approximately 1% to 2% of the population.^1^ IDD comprises conditions characterized by altered intellectual, emotional, and/or physical developmental trajectories that begin before age 18 years and may involve many body systems.^2^

People with IDDs experience worse health and pregnancy outcomes than the general population.^3,4^ Rubenstein et al^5^ found approximately 50% increased risks of preterm birth (PTB) and small-for-gestational-age (SGA) birth associated with maternal IDDs in 2007 to 2016 Wisconsin Medicaid data. In Ontario, Canada (2012-2019), infants of mothers with IDDs had an approximately 50% to 80% increased risk of PTB and neonatal intensive care unit (NICU) admission compared with infants born to mothers without a disability.^6^ Akobirshoev et al,^7^ using 2007 to 2011 Nationwide Inpatient Sample data, also found a 2-fold greater risk of PTB among infants born to individuals without IDDs.

Although these early studies highlighted disparities in adverse birth outcomes, limitations reduced clinical application. Because IDDs among birthing people are rare, studies aggregated IDD subtypes despite distinct pathophysiologic mechanisms and comorbidity profiles. Most analyses adjusted for potential mediators (variables on the causal pathway between maternal IDDs and infant outcomes), thereby underestimating true effects and obscuring intervention opportunities.^8^ To expand on this literature, we examined associations of maternal IDD subtypes (ASD, ID, CP, chromosomal differences, and an “other” category) with NICU admission, SGA birth, and prematurity. Using causal mediation analyses, we estimated the proportion of the excess risk associated with modifiable maternal characteristics, providing evidence-based targets for intervention.

## Methods

### Study Sample

We conducted a retrospective cohort study using singleton live birth data from January 1, 2007, to December 31, 2021, from the Study of Outcomes in Mothers and Infants (SOMI). The methods used to construct SOMI have been previously documented.^9^ In brief, vital statistics records were linked with hospital discharge, emergency department, and ambulatory surgery data for the person giving birth and the infant and maintained by the California Department of Health Care Access and Information for a near-complete collection of California births. For each linked mother-child dyad, hospital records include the year prior to delivery (maternal) and the year after delivery (maternal and child). SOMI also contains data for fetal deaths occurring after 22 weeks’ gestation. The study was approved by the Committee for the Protection of Human Subjects within the Health and Human Services Agency of the state of California and by the institutional review board at the University of California, San Diego. A waiver of informed consent was granted by the institutional review board bodies because the research posed minimal risk to participants, did not adversely affect the rights of the participants, and was impractical given the number of individuals. This study follows the Strengthening the Reporting of Observational Studies in Epidemiology (STROBE) reporting guideline for cohort studies and the Guideline for Reporting Mediation Analyses of Randomized Trials and Observational Studies (AGReMA)^10^ recommendations. Throughout this article, we use maternal, mother, and women to align with epidemiologic terminology, while recognizing that pregnancy is not limited to cisgender women.

### Exposure, Outcomes, and Covariates

Maternal IDD was identified via *International Classification of Diseases, Ninth Revision* (*ICD-9*) and *International Statistical Classification of Diseases and Related Health Problems, Tenth Revision* (*ICD-10*) codes from maternal records (eTable 1 in Supplement 1). Based on previous literature,^4^ IDDs were categorized into 5 groups: ASD, CP, ID, chromosomal differences, and other IDDs. The other IDDs group consists mainly of IDD conditions caused by congenital differences or teratogenic exposures (see eTable 1 in Supplement 1 for full list). IDD subtypes were not mutually exclusive, and pregnant people with multiple IDD diagnoses contributed to each model corresponding to their respective conditions. However, there was little overlap between groups.

Adverse infant outcomes, extracted from the California Vital Statistics birth cohort files, included NICU admission, SGA birth, and birth occurring at less than 37 weeks (PTB) or less than 32 weeks of gestation (very PTB). Demographic characteristics collected from birth certificate records included maternal age at birth, year of birth, maternal educational level (dichotomized as <12 years vs ≥12 years), maternal Special Supplemental Nutrition Program for Women, Infants, and Children (WIC) enrollment status at birth, health insurance payer for birth, adequacy of prenatal care,^11^ maternal prepregnancy body mass index (BMI [calculated as weight in kilograms divided by height in meters squared]; dichotomized into <25 vs ≥25), prepregnancy hypertension, and prepregnancy diabetes. Maternal race and ethnicity, which was self-reported, was also collected from birth certificate records and included 5 categories: Asian, Hispanic, non-Hispanic Black, non-Hispanic White, and other (American Indian or Alaska Native, Hawaiian or Pacific Islander, self-reported other race, ≥2 races, and not stated or unknown). Because race and ethnicity are associated with both the mediators and the infant outcomes studied, we adjusted for self-reported race and ethnicity throughout the mediation models. Depression, anxiety, bipolar disorder, schizophrenia, and epilepsy diagnoses were collected from maternal hospital records. Smoking status was ascertained from both birth certificate records and *ICD-9* and *ICD-10* diagnoses documented in discharge records. Diagnosis codes used to identify these conditions are listed in eTable 1 in Supplement 1.

### Statistical Analysis

Statistical analyses were completed on February 19, 2026. We created six 30:1 (no IDD:IDD[subtype]) matched samples: 1 for IDDs overall and 1 for each IDD subtype. We matched on maternal age at birth (≤35 years vs >35 years) and year of delivery. Although not traditional confounders (they did not cause IDDs), they may be associated with diagnostic trends and were therefore included. From the matched cohorts, we estimated adjusted risk ratios (ARRs) and their corresponding 95% CIs via modified Poisson regression models. Standard errors were adjusted for the inclusion of multiple births from the same mothers using a sandwich estimator.^12^ Models were repeated for each outcome. We completed probabilistic and deterministic quantitative bias analyses to estimate the degree of IDD misclassification needed to meaningfully change conclusions. The description of these analyses and underlying assumptions is included in the eMethods in Supplement 1.

We conducted single and joint causal mediation analyses. Causal mediation analyses and the assumptions used have been described previously.^13,14^ Causal mediation analyses estimate effects only if the assumed causal relationships among the exposure, mediators, and outcome hold. The study design of causal mediation itself, however, does not establish causality. Causal mediation analysis can decompose the total effect of an exposure on an outcome into 2 components: the indirect effect, which operates through a mediator, and the direct effect, which captures all remaining pathways. The direct effect can be thought of as the effect of the exposure that would persist even if the mediator were held constant, representing the influence of the exposure on the outcome through mechanisms other than the mediators in question. Marginal structural models and a counterfactual (potential outcomes) framework were used to estimate the direct effects, indirect effects, total effects, and proportions of the total effect of IDDs or IDD subtype accounted for by the mediator. For simplicity, this last estimate is referred to as the *proportion mediated* in the text. The total effect is the effect of the exposure on the outcome, including both direct and indirect effect pathways. If the total and indirect effects were in different directions, the proportion mediated was not estimated to avoid negative percentage point estimates. In addition, proportions mediated exceeding 100% or less than 0% are mathematically possible (see formulas in eTables 5-8 in Supplement 1) when effects are imprecise or unstable and/or when the total and indirect effects point in opposite directions in some bootstrap samples. We used marginal structural models to allow for possible mediator-outcome confounders to be caused by IDDs or IDD subtype (ie, a mediator that is also a confounder). Prior to performing mediation analyses, cell sizes were examined to ensure that all cells had 5 or more pregnancies after IDDs or IDD subtype and outcome were stratified by the mediator. Joint mediation analyses were completed for each outcome. Each joint mediation model included all mediators that met cell size inclusion for the single mediation models. We did not include tests of interaction in the mediation analyses due to sample size limitations.

Mediators of interest were selected based on subject matter expertise, data availability, and ability to intervene on them. These mediators were prenatal tobacco use, adequacy of prenatal care, prepregnancy chronic hypertension, prepregnancy diabetes, maternal BMI, epilepsy, depression or anxiety, and bipolar disorder or schizophrenia. In all mediation models, maternal age and year of delivery were included as exposure-outcome confounders, while covariates included as confounders of the mediator outcome were maternal educational level, rurality, maternal race and ethnicity, year of delivery, maternal age, and WIC enrollment (included as a postexposure confounder). In models in which NICU and SGA birth were the outcomes, PTB was additionally included as a mediator. For the NICU and SGA birth outcomes, the following variables were included as postexposure confounders in single-mediation models: maternal BMI, preexisting diabetes, preexisting hypertension, depression or anxiety diagnoses, maternal epilepsy, bipolar disorder or schizophrenia diagnoses, and maternal tobacco use during pregnancy.

All analyses other than the mediation analyses were performed using StataNow/SE, version 19.5 (StataCorp LLC).^15^ Causal mediation analysis was performed using the R user-created program CMAverse in R, version 4.5.1 (R Project for Statistical Computing).^16^

## Results

### Cohort Characteristics

**Figure 1.**
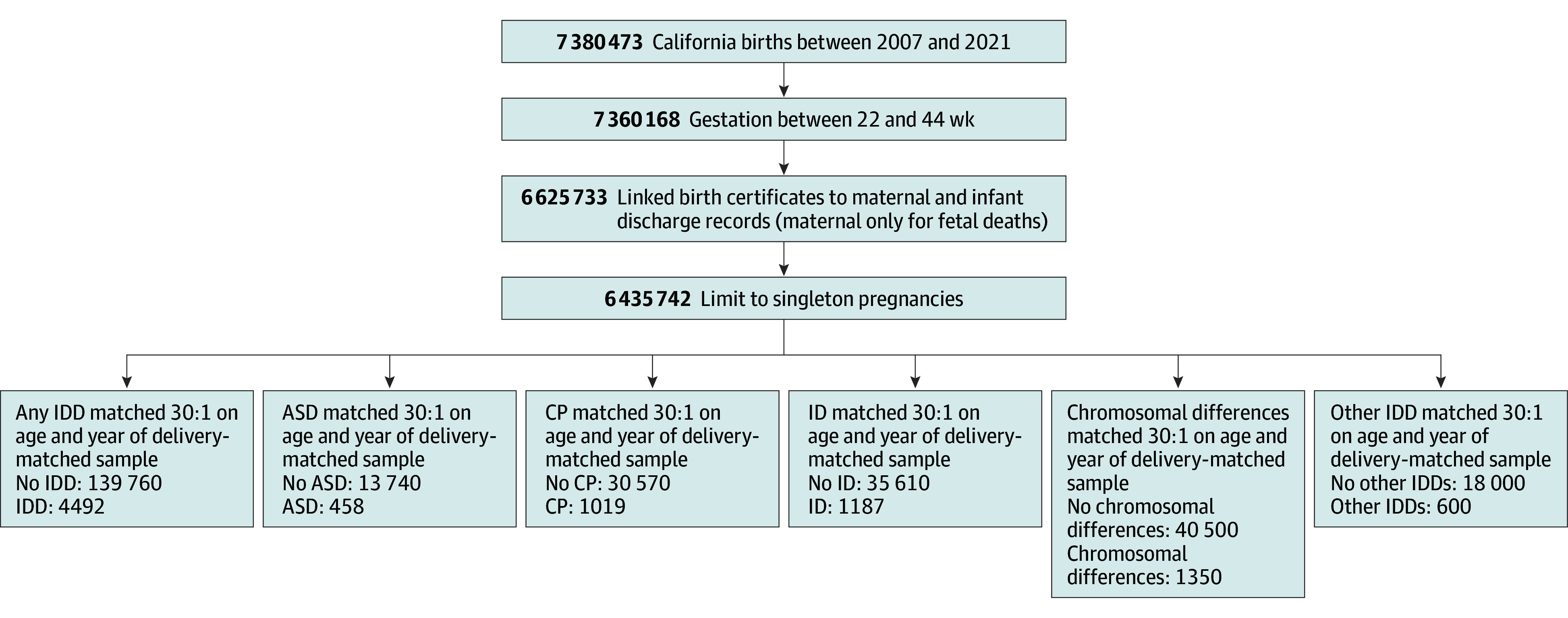
Flow Diagram ASD indicates autism spectrum disorder; CP, cerebral palsy; ID, intellectual disability; IDD, intellectual and developmental disability; and other IDD, fetal alcohol syndrome, tuberous sclerosis, and various congenital malformations noted in eTable 1 in Supplement 1. Counts include fetal deaths, and IDD subtypes are not mutually exclusive.

Of 6 435 742 singleton births, 4492 were to mothers with IDDs (mean [SD] maternal age at birth, 29 [7] years; 259 Asian [6%], 1859 Hispanic [41%], 427 non-Hispanic Black [10%], 1584 non-Hispanic White [35%], and 363 other race or ethnicity [8%]) (Figure 1 and Table 1). In the overall matched cohort, the mean (SD) maternal age at birth was 30 (6) years, and the largest race and ethnicity group was Hispanic women (48% [65 537 of 134 760]). Of mothers with IDDs, 458 had an ASD diagnosis, 1019 had a CP diagnosis, 1187 had an ID diagnosis, 1350 had a chromosomal difference diagnosis, and 600 had other IDD diagnoses. Forty-nine people had both ID and CP diagnoses, 33 people had both ID and ASD diagnoses, 20 people had both ID and chromosomal differences, and 18 people had other combinations of IDD diagnoses. The proportion of singleton births with a maternal IDD diagnosis increased over the analytic period (5 of 10 000 live births in 2007; 9 of 10 000 live births in 2021). People with IDDs in comparison with people without IDDs were more likely to be non-Hispanic White, have a public payer at birth, be enrolled in WIC at birth, have prepregnancy chronic hypertension, have diabetes, have a BMI of 25 or more, and to use tobacco during pregnancy (Table 1). They were also more likely to have anxiety, depression, bipolar disorder, schizophrenia, and epilepsy diagnoses.

**Table 1.** Demographic and Clinical Factors Among People With IDDs and Those Without During Pregnancy

Factor	No IDDs (n = 134 760)^a^	IDDs (n = 4492)
Infant outcomes		
NICU admission, No. (%)	6818 (5)	628 (14)
Small for gestational age birth, No. (%)	11 822 (9)	614 (14)
Very preterm birth (<32 wk), No. (%)	1142 (1)	122 (3)
Preterm birth (<37 wk), No. (%)	9202 (7)	717 (16)
Maternal demographics		
Maternal age at birth, mean (SD), y	30 (6)	29 (7)
Race and ethnicity, No. (%)		
Asian	19 436 (14)	259 (6)
Hispanic	65 537 (49)	1859 (41)
Non-Hispanic Black	6174 (5)	427 (10)
Non-Hispanic White	36 192 (27)	1584 (35)
Other^b^	7421 (6)	363 (8)
Year of birth, mean (SD)	2014 (4)	2014 (4)
Maternal educational level at birth, No. (%)		
<12 y	22 440 (17)	845 (19)
≥12 y	106 195 (79)	3338 (74)
Missing	6125 (5)	309 (7)
WIC status, No. (%)		
No WIC	70 847 (53)	1840 (41)
WIC	62 838 (47)	2585 (58)
Missing	1075 (1)	67 (1)
Payer, No. (%)		
Private	73 508 (55)	1833 (41)
Public	61 236 (45)	2630 (59)
Other	16 (0.01)	29 (1)
Rurality, median (IQR)^c^	1 (1-2)	1 (1-3)
Maternal IDD subtypes, No. (%)^d^		
Autism spectrum disorder	NA	458 (10)
Intellectual disability	NA	1187 (26)
Cerebral palsy	NA	1019 (23)
Chromosomal differences	NA	1350 (30)
Other IDD^e^	NA	600 (13)
Maternal clinical characteristics, No. (%)		
Adequate prenatal care (dichotomized Kotelchuck index)		
Inadequate or intermediate	33 235 (25)	1241 (28)
Adequate or better than adequate	97 298 (72)	3002 (67)
Missing	4227 (3)	249 (6)
BMI (dichotomized)		
Underweight or normal (<25)	65 723 (49)	1905 (42)
Overweight or obese (≥25)	63 602 (47)	2326 (52)
Missing	5435 (4)	261 (6)
Preexisting hypertension	3004 (2)	281 (6)
Preexisting diabetes	1688 (1)	156 (3)
Tobacco use during pregnancy	560 (0.4)	113 (3)
Anxiety or depression diagnosis	5154 (4)	709 (16)
Bipolar disorder	530 (0.4)	303 (7)
Schizophrenia	90 (0.07)	181 (4)
Bipolar disorder or schizophrenia	578 (0.4)	382 (9)
Epilepsy	464 (0.3)	316 (7)

Abbreviations: BMI, body mass index (calculated as weight in kilograms divided by height in meters squared); IDD, intellectual and developmental disabilities, NA, not applicable; NICU, neonatal intensive care unit; WIC, USDA’s Special Supplemental Nutrition Program for Women, Infants, and Children.

^a^ Matched 30:1 on maternal age (≤35 years vs >35 years) and year of delivery.

^b^ Other race category included American Indian or Alaska Native, Hawaiian or Pacific Islander, self-reported other racial or ethnic group, 2 or more races, and not stated or unknown.

^c^ Rurality determined by Federal Information Processing Standards urban/rural county code continuum, where 1 indicates most urban and 6 indicates most rural.

^d^ IDD subtypes are not mutually exclusive.

^e^ Other IDDs composed of fetal alcohol syndrome, tuberous sclerosis, and various congenital malformations (eTable 1 in Supplement 1).

When IDDs were stratified by subtype, many prepregnancy characteristics varied (eTable 2 in Supplement 1). People with chromosomal differences were oldest at birth (mean [SD] age, 32 [7] years), while people with ASD were youngest (mean [SD] age, 26 [6] years). People with CP had the highest proportion (82% [840 of 1019]) completing at least 12 years of education, while people with ID had the lowest (62% [736 of 1187]). Adequate prenatal care receipt was highest among people with chromosomal differences (75% [1009 of 1350]) or other IDDs (75% [451 of 600]) and lowest among people with ID (52% [621 of 1187]). Anxiety and/or depression diagnoses were most common among people with ASD (34% [155 of 458]) and ID (23% [271 of 1187]) and least common among people with chromosomal differences (9% [127 of 1350]). Bipolar and/or schizophrenia disorders were also most common among people with ASD (21% [95 of 458]) and ID (21% [252 of 1187]) and least common among people with chromosomal differences (1% [<11 of 1350]). Epilepsy showed a similar pattern, but people with CP additionally had high levels (CP, 13% [129 of 1019]; ID, 12% [142 of 1187]; ASD, 9% [39 of 458]).

### Infant Outcomes

The incidences of all adverse infant outcomes were increased among infants born to mothers with IDDs vs infants born to mothers without IDDs. Per 100 births among births to women with IDDs vs births to women without IDDs, there were 9 more NICU admissions (95% CI, 7.90-9.94), 5 more infants born SGA (95% CI, 3.88-5.91), 9 more preterm infants (95% CI, 8.05-10.21), and 2 more very preterm infants (95% CI, 1.39-2.35) (Table 2). In the matched sample, infants born to mothers with IDDs had higher risks of NICU admission (14% [628 of 4492] vs 5% [327 345 of 6 426 048]; ARR, 2.76 [95% CI, 2.56-2.98]), SGA birth (14% [614 of 4492] vs 9% [569 146 of 6 426 048]; ARR, 1.56 [95% CI, 1.44-1.68]), PTB (16% [717 of 4492] vs 7% [452 177 of 6 426 048]; ARR, 2.34 [95% CI, 2.18-2.51]), and very PTB (3% [122 of 4492] vs 1% [62 290 of 6 426 048]; ARR, 3.20 [95% CI, 2.66-3.86]) compared with infants born to mothers without IDD (Table 2 and Table 3). All subtypes of IDDs (Table 3) had increased risk of NICU admission, SGA birth, PTB, and very PTB. Infants born to mothers with CP and ASD diagnoses tended to have the lowest risk ratios for all adverse infant outcomes (ARR range for mothers with ASD, 1.41-2.59; ARR range for mothers with CP, 1.46-2.82). Infants born to mothers in the other IDDs category had the highest risk ratios for most outcomes (ARR range, 1.70-5.06).

**Table 2.** Infant Outcomes in California Study of Outcomes in Mothers and Infants, by Maternal IDD Status, 2007-2021

Infant outcome	No. (%)							
	No IDD (n = 6 426 048)	IDD (n = 4492)	Autism spectrum disorder (n = 458)	Cerebral palsy (n = 1019)	ID (n = 1187)	Chromosomal differences (n = 1350)	Other IDD (n = 600)^a^
NICU admission	327 345 (5)	628 (14)	59 (13)	83 (8)	189 (16)	230 (17)	93 (16)
Small-for-gestational-age birth	569 146 (9)	614 (14)	59 (13)	130 (13)	196 (17)	173 (13)	90 (15)
Preterm birth							
<32 wk	62 290 (1)	122 (3)	<11 (<2)^b^	24 (2)	33 (3)	39 (3)	26 (4)
<37 wk	452 177 (7)	717 (16)	60 (13)	158 (16)	203 (17)	212 (16)	104 (17)

Abbreviations: ID, intellectual disability; IDD, intellectual and developmental disability; NICU, neonatal intensive care unit.

^a^ Other IDD includes fetal alcohol syndrome, tuberous sclerosis, and various congenital malformations (eTable 1 in Supplement 1); counts include fetal deaths and IDD subtypes are not mutually exclusive.

^b^ To reduce probability of reidentification of data, we are required to mask counts of fewer than 11.

**Table 3.** Association of IDDs and IDD Subtypes With Adverse Birth Outcomes in California, 2007-2021

IDD or subtype^b^	Adjusted risk ratio (95% CI)^a^			
	NICU admission	SGA	PTB (<37 wk)	Very PTB (<32 wk)
Any IDD (n = 139 252)	2.76 (2.56-2.98)	1.56 (1.44-1.68)	2.34 (2.18-2.51)	3.20 (2.66-3.86)
Autism spectrum disorder (n = 14 198)	2.59 (2.02-3.32)	1.41 (1.10-1.80)	2.00 (1.56-2.57)	2.04 (0.95-4.36)
Intellectual disability (n = 36 797)	2.95 (2.57-3.39)	1.80 (1.57-2.06)	2.64 (2.31-3.01)	3.50 (2.45-4.99)
Cerebral palsy (n = 31 589)	1.71 (1.37-2.14)	1.46 (1.23-1.72)	2.33 (1.99-2.73)	2.82 (1.84-4.33)
Chromosomal differences (n = 41 850)	3.20 (2.82-3.62)	1.51 (1.31-1.75)	2.10 (1.84-2.38)	2.26 (1.64-3.12)
Other IDD (n = 18 600)^c^	2.99 (2.44-3.65)	1.70 (1.39-2.09)	2.56 (2.12-3.08)	5.06 (3.37-7.61)

Abbreviations: IDD, intellectual and developmental disability; NICU, neonatal intensive care unit; PTB, preterm birth; SGA, small for gestational age.

^a^ 1:30 Matching was performed on maternal age at birth (≤35 years vs >35 years) and year of delivery for all samples; exponentiated coefficients; 95% CIs in parentheses; and standard errors adjusted for multiple pregnancies from same person.

^b^ Total analytic sample size of matched sample.

^c^ Other IDD includes fetal alcohol syndrome, tuberous sclerosis, and various congenital malformations (eTable 1 in Supplement 1).

Our results were more robust to reduced sensitivity than reduced specificity in quantitative bias analyses (eTables 3 and 4 in Supplement 1). In brief, in the probabilistic bias analysis, after accounting for misclassification of IDDs, the observed associations between maternal IDDs and adverse infant outcomes became more pronounced (eTable 4 in Supplement 1). For any IDD, the median ARRs were 5.90 (95% CI, 4.16-62.88) for NICU admission, 1.91 (95% CI, 1.71-2.69) for SGA birth, 4.14 (95% CI, 3.15-44.25) for PTB, and 8.41 (95% CI, 5.30-115.88) for very PTB. These findings indicate that even after accounting for reduced exposure sensitivity, IDDs remain strongly associated with adverse birth outcomes. Contrastingly, if we assume reduced specificity of the exposure among births in which the infant had the outcome, results may be sensitive to small reductions in specificity.

### Causal Mediation Analyses

#### Single-Mediation Analysis

**Figure 2.**
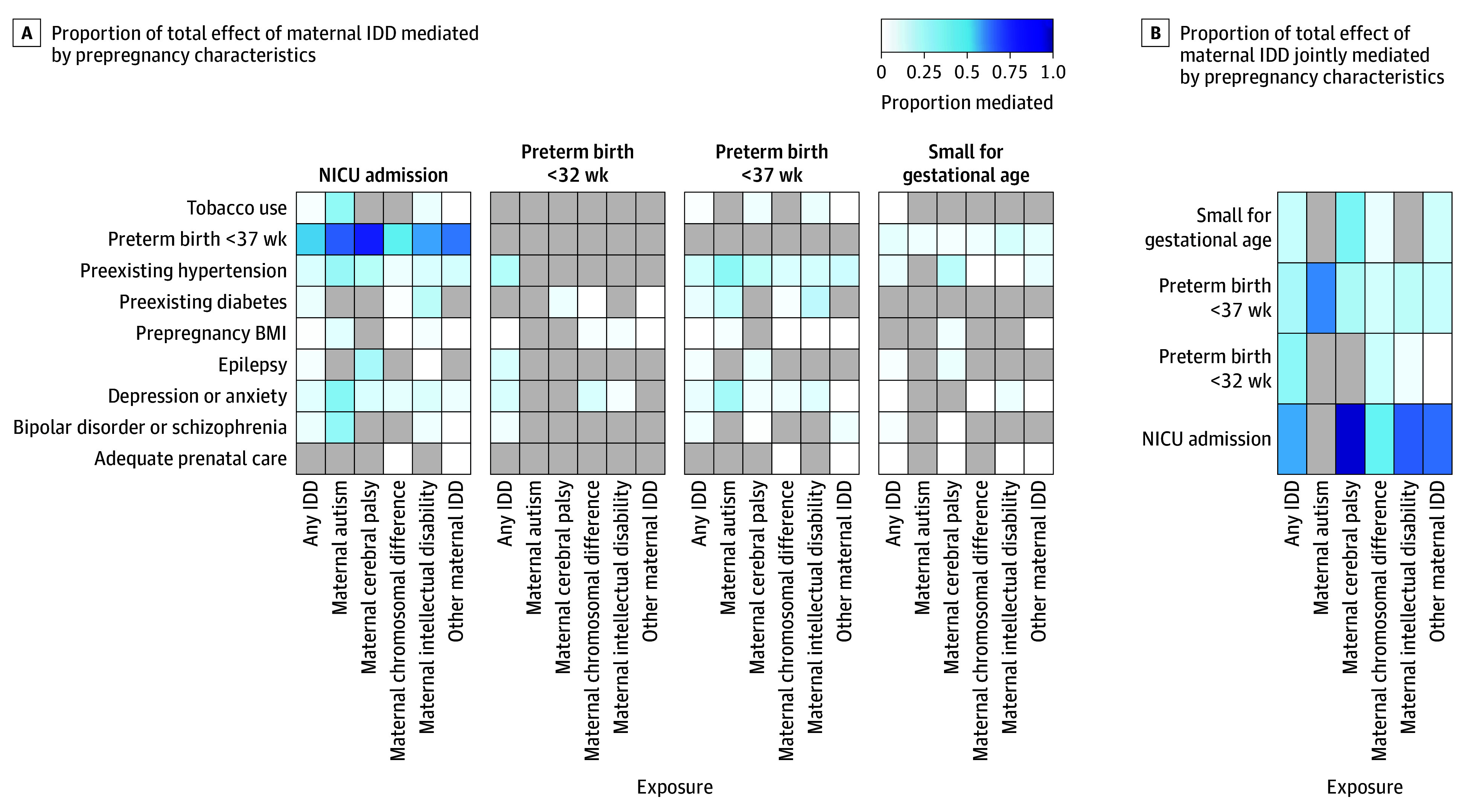
Heat Maps of Proportion of Total Effect of Maternal Intellectual and Developmental Disabilities (IDDs) Mediated by Maternal Prepregnancy Characteristics Mediators are included if (when sample is stratified by exposure, outcome, and mediator) all cells have 5 or fewer individuals. Body mass index (BMI) was dichotomized as underweight or normal BMI vs overweight or obese BMI. Mediation models for all exposure mediator combinations (except for IDD → preterm birth [PTB] <37 weeks → neonatal intensive care unit [NICU]; arrows indicate a causal path from IDD to PTB to NICU admission) were adjusted for maternal educational level (<12 years vs ≥12 years), rurality (Federal Information Processing Standards urban/rural county code continuum, where 1 indicates most urban and 6 indicates most rural), maternal race and ethnicity, year of delivery (categorical), maternal age at delivery (continuous), and maternal USDA’s Special Supplemental Nutrition Program for Women, Infants, and Children (WIC) enrollment. Mediation models (IDD → PTB <37 weeks → NICU) were adjusted for maternal educational level (<12 years vs ≥12 years), rurality (California), maternal race and ethnicity, year of delivery (categorical), maternal age at delivery (continuous), maternal WIC enrollment, maternal BMI (underweight or normal vs overweight or obese), maternal preexisting diabetes, maternal preexisting hypertension, maternal anxiety or depression, maternal epilepsy, maternal bipolar disorder or schizophrenia, and maternal tobacco use during pregnancy. The list of mediators included in each joint mediation can be found in eTables 5 to 8 in Supplement 1.

The strongest single mediators (Figure 2A; eTables 5-8 in Supplement 1) between IDDs and the subtypes with adverse outcomes were preexisting hypertension and mental health conditions, particularly for NICU admissions and PTB. PTB, when considered as a mediator in the NICU admissions models, was also a particularly strong mediator. Estimates were imprecise, with heterogeneity in the strength of the mediator by IDD subtype and outcome. The strongest single mediator proportion mediated, outside of PTB-mediated NICU models, was observed for NICU admission due to maternal depression or anxiety among people with ASD (30.6% [95% CI, –58.0% to 219.7%]). In contrast, 7.1% (95% CI, 4.3%-10.7%) of the excess risk of chromosomal differences for NICU admission was mediated through maternal depression or anxiety. In the IDD subtype models for PTB-mediated NICU risk, proportion mediated estimates were generally above 50%, with the highest being among mothers with CP (76.0% [95% CI, 59.9%-133.9%]).

#### Joint Mediation Analysis

Results for multiple-mediation analyses (Figure 2B) varied greatly by IDDs or IDD subtype and outcome. When NICU admission was the outcome, all proportion mediated estimates for all IDD subtypes were high (>40%) (Figure 2B). Nearly all the excess risk of maternal CP for NICU admission (96.1% [95% CI, 70.0%-161.0%]) was explained by maternal prenatal characteristics (PTB, prepregnancy BMI, preexisting hypertension, depression or anxiety, epilepsy, and prenatal care). Sixty percent of the excess risk of maternal ASD for PTB was mediated by select prepregnancy characteristics (adequacy of prenatal care, prepregnancy BMI, preexisting diabetes, preexisting hypertension, and depression or anxiety). Nearly one-third of the excess risk of maternal CP for SGA birth (34%) and over one-fourth of any IDD for very PTB (28.1% [95% CI, 17.6%-45.7%]) was mediated by prepregnancy characteristics.

## Discussion

In this large California administrative birth cohort, we expand prior findings by disaggregating IDDs into subtypes and examining mediating factors associated with the persistently observed excess risk of adverse outcomes. Infants of women with IDDs had higher risks of NICU admission, prematurity, and SGA birth, with all IDD subtypes having increased risks compared with births from people without IDDs. Estimates were lowest for infants born to mothers with ASD or CP and highest for those in the other IDD category and those with chromosomal differences. Results were generally robust to misclassification of the exposure. The excess risk of some IDD subtypes was partially explained by maternal comorbidities such as prepregnancy chronic hypertension and mental health diagnoses.

Our findings of an increased risk of select adverse birth outcomes associated with maternal IDDs is consistent with the previous literature.^3,5,6,7^ Replicating previous findings from the US and Canada,^3,5,7,17,18^ our sample of birthing parents with IDDs had higher proportions of birthing parents with a BMI of 25 or more, chronic diseases, and signs of mental distress than birthing parents without IDDs. These factors are known to be associated with adverse infant outcomes,^19,20,21^ motivating our mediation analyses.

Increased risks among infants born to individuals with IDDs underscore the need for targeted interventions. Mediation analyses suggest that much of the excess risk is explained by prepregnancy chronic hypertension, epilepsy, and mental health conditions. The American College of Obstetrics and Gynecology recommends numerous preconception lifestyle interventions, which include managing chronic health conditions such as hypertension and psychiatric illnesses.^22^ Previous studies have identified several effective interventions, including following a healthy diet and engaging in regular exercise (≥150 minutes/week).^23^ Given the complex health needs of individuals with IDDs, multidisciplinary preconception care, including mental health and neurology, may improve infant outcomes.

Future research should replicate findings in other data sources and examine pharmacologic treatments for comorbidities as potential mediators. Some antiepileptics, psychotropics, and antihypertensives are not recommended during pregnancy and may increase the risk of select outcomes. Barriers to preconception interventions or care for women with IDDs may also be a focus of future work.

### Strengths and Limitations

The strengths of this study include that this population-based study of births in California (2007-2021) is the largest to date examining infant outcomes among birthing parents with IDDs. The design reduced selection bias and improved generalizability beyond Medicaid-based studies. The large sample and extensive covariate set enabled nuanced analyses by IDD subtype and quantification of mediated pathways.

There are also some limitations to the study. Disentangling the effects of IDD-related conditions from their treatments is challenging, particularly for epilepsy and mental health disorders. In addition, our reliance on delivery records may have oversampled individuals with IDDs with extensive support needs,^24^ leading to findings that are generalizable only to the most severe cases. Similarly, there are inherent limitations in using diagnosis codes to identify people with disabilities. Disability, as captured by diagnosis codes, may not fully or accurately capture the practical limitations and experiences of a person with a disability.^24^ Based on bias analyses, reductions in sensitivity of the exposure ascertainment would need to be quite strong to alter our findings. In addition, for IDD conditions that can be diagnosed in utero or around birth (eg, Down syndrome), some maternal records may reflect infant diagnoses. Although our results would be sensitive to small reductions in IDD exposure specificity if it varied by outcome status (eMethods in Supplement 1), future work should investigate this potential misclassification in administrative records. Furthermore, we lacked information on reduced conception and fetal deaths that occurred prior to 22 weeks, which can result in selection bias.^25^ However, the present study includes fetal deaths that occurred on or after 22 weeks’ gestation, which is an advantage over previous literature.

## Conclusions

This cohort study found that infants born to mothers with IDDs were at higher risks of adverse birth outcomes, including NICU admission, PTB, and SGA birth, with risks varying by IDD subtype. Because preexisting chronic hypertension, epilepsy, and mental health conditions mediated these risks, individuals with IDDs and their offspring may benefit from focused screening and interventions for these conditions.

## Notes

Supplement 1. **eTable 1. ***International Classification of Disease* (*ICD*) Codes Used to Identify Study Diagnoses **eMethods. **Quantitative Bias Analysis Methods
eReferences.
**eTable 2. **Demographic and Clinical Characteristics of IDD Sample, by IDD Subtype **eResults. **Quantitative Bias Analyses Results **eTable 3. **Exposure Sensitivity Among Non-Cases Needed to Attenuate Risk Ratio Estimates to 1.0, by IDD Type and Outcome **eTable 4. **Results of Varying Sensitivity of Exposure Classification Between 7%-17%, Assuming Specificity is 100% and Misclassification of Exposure is Nondifferential **eTable 5.** Risk Ratios for Total, Direct and Indirect Effects and Proportion Mediated, by IDD/IDD Subtype, Outcome, and Mediator – NICU Admission **eTable 6.** Risk Ratios for Total, Direct and Indirect Effects and Proportion Mediated, by IDD/IDD Subtype, Outcome, and Mediator – Preterm Birth <32 Weeks **eTable 7. **Risk Ratios for Total, Direct and Indirect Effects and Proportion Mediated, by IDD/IDD Subtype, Outcome, and Mediator – Preterm Birth <37 Weeks **eTable 8.** Risk Ratios for Total, Direct and Indirect Effects and Proportion Mediated, by IDD/IDD Subtype, Outcome, and Mediator – Small for Gestational Age Infant

Supplement 2.
Data Sharing Statement
