# Supplementary material for: Maternal Intellectual and Developmental Disabilities and Infant Outcomes
Source: JAMA Netw Open. 2026 May 27;9(5):e2615005. doi: 10.1001/jamanetworkopen.2026.15005 (PMC13216985; doi:10.1001/jamanetworkopen.2026.15005)
Supplement: Supplement 1. — eTable 1. International Classification of Disease (ICD) Codes Used to Identify Study Diagnoses eMethods. Quantitative Bias Analysis Methods eReferences. eTable 2. Demographic and Clinical Characteristics of IDD Sample, by IDD Subtype eResults. Quantitative Bias Analyses Results eTable 3. Exposure Sensitivity Among Non-Cases Needed to Attenuate Risk Ratio Estimates to 1.0, by IDD Type and Outcome eTable 4. Results of Varying Sensitivity of Exposure Classification Between 7%-17%, Assuming Specificity is 100% and Misclassification of Exposure is Nondifferential eTable 5. Risk Ratios for Total, Direct and Indirect Effects and Proportion Mediated, by IDD/IDD Subtype, Outcome, and Mediator – NICU Admission eTable 6. Risk Ratios for Total, Direct and Indirect Effects and Proportion Mediated, by IDD/IDD Subtype, Outcome, and Mediator – Preterm Birth <32 Weeks eTable 7. Risk Ratios for Total, Direct and Indirect Effects and Proportion Mediated, by IDD/IDD Subtype, Outcome, and Mediator – Preterm Birth <37 Weeks eTable 8. Risk Ratios for Total, Direct and Indirect Effects and Proportion Mediated, by IDD/IDD Subtype, Outcome, and Mediator – Small for Gestational Age Infant [file jamanetwopen-e2615005-s001.pdf]

Supplementary Online Content

Psaras C, Ryu RH, Baer R, et al. Maternal intellectual and developmental disabilities and infant outcomes. *JAMA Netw Open.* 2026;9(5):e2615005. doi:10.1001/jamanetworkopen.2026.15005

eTable 1. *International Classification of Disease* (*ICD*) Codes Used to Identify Study Diagnoses

eMethods. Quantitative Bias Analysis Methods

eReferences.

eTable 2. Demographic and Clinical Characteristics of IDD Sample, by IDD Subtype

eResults. Quantitative Bias Analyses Results

eTable 3. Exposure Sensitivity Among Non-Cases Needed to Attenuate Risk Ratio Estimates to 1.0, by IDD Type and Outcome

eTable 4. Results of Varying Sensitivity of Exposure Classification Between 7%-17%, Assuming Specificity is 100% and Misclassification of Exposure is Nondifferential

eTable 5. Risk Ratios for Total, Direct and Indirect Effects and Proportion Mediated, by IDD/IDD Subtype, Outcome, and Mediator – NICU Admission

eTable 6. Risk Ratios for Total, Direct and Indirect Effects and Proportion Mediated, by IDD/IDD Subtype, Outcome, and Mediator – Preterm Birth <32 Weeks

eTable 7. Risk Ratios for Total, Direct and Indirect Effects and Proportion Mediated, by IDD/IDD Subtype, Outcome, and Mediator – Preterm Birth <37 Weeks

eTable 8. Risk Ratios for Total, Direct and Indirect Effects and Proportion Mediated, by IDD/IDD Subtype, Outcome, and Mediator – Small for Gestational Age Infant

This supplementary material has been provided by the authors to give readers additional information about their work.

© 2026 Psaras C et al. *JAMA Network Open.*

eTable 1. *International Classification of Disease* (*ICD*) Codes Used to Identify Study Diagnoses

Diagnosis	ICD-9	ICD-10	Notes
Intellectual disability	317, 318, 319,	F70, F71, F72, F73, F78, F79	
Autism Spectrum Disorder	299	F84.0, F84.1, F84.3, F84.4, F84.5, F84.8, F84.9	
Cerebral Palsy	343	G80	
Chromosomal Differences	758.0, 758.3, 758.5, 758.82, 758.83, 758.84, 758.85, 758.86, 758.87, 758.88, 758.89, 758.9, 759.83	Q90, Q92.2, Q92.3, Q92.4, Q92.5, Q92.8, Q92.9, Q93, Q99.2, Q99.8	Includes: Down Syndrome, Fragile X syndrome, autosomal deletion syndromes, partial trisomy, duplications with other complex rearrangements, and other conditions due to chromosomal anomalies
Other intellectual and developmental disabilities	760.71, 760.77, 759.5, 759.821, 759.827, 759.828, 759.874, 759.875, 759.891, 759.892, 759.893, 759.894, 759.895, 759.896, 759.897, 759.898, 759.899	Q86.0, Q85.1, Q86.1, Q87.1, Q87.2, Q87.3, Q87.8	Includes: Fetal alcohol syndrome, tuberous sclerosis, congenital malformation syndromes predominantly associated with short stature, congenital malformation syndromes predominantly involving limbs, congenital malformation syndromes involving early overgrowth, and other specified congenital malformation syndromes
Gestational hypertension	642.3, 642.4, 642.5, 642.6	O13, P00.0 (infant), O14.0, O14.1, O14.2, O14.9, O15	
Anxiety	300	F40, F41	
Depression	296.2, 296.3, 311	F32, F33	
Bipolar disorder	296.0, 296.1, 296.4, 296.5, 296.6, 296.7, 296.8	F31	
Schizophrenia	295	F20	
Preexisting diabetes (Types I and II)	648.0, 249, 250	O24.0, O24.1, O24.2, O24.3, E10, E11, E12, E13, E14,	

© 2026 Psaras C et al. *JAMA Network Open.*

		P70.1 (infant), O24.4, P70.0 (infant)
Preexisting hypertension	642.0, 642,1, 642.1, 642.2, 642.7	O10, O11
Epilepsy	345	G40
Tobacco use during pregnancy	649.0, 305.1	F17, P04.2 (infant), Z72.0

© 2026 Psaras C et al. *JAMA Network Open.*

eMethods. Quantitative Bias Analysis Methods

Reduced Sensitivity

To address this potential misclassification, probabilistic and deterministic quantitative bias analyses were conducted. Deterministic analyses assessed the degree of reduced sensitivity required for IDD and its subtypes to substantially alter the conclusions drawn from our original analyses. In the deterministic bias analysis, we estimated the sensitivity of IDD classification among non-cases (differential) needed to attenuate RR estimates to the null (RR=1.0). Results are in Table S3.

In the probabilistic quantitative bias analyses, we uniformly varied the sensitivity of exposure classification between 7% and 17% over 1,000 repetitions. IDD and IDD subtype misclassification in the probabilistic analyses were assumed not to vary by outcome status (non-differential misclassification). These values were chosen based on the sensitivity of autism spectrum disorder (ASD) diagnosis in healthcare claims data in a previous study.[1] In the probabilistic bias analyses, we estimated the median and 2.5th and 97.5th percentile RRs from the 1,000 repetitions. Results are in Table S4.

Reduced Specificity

Some mothers may have been erroneously classified as having IDD if the chromosomal difference was actually present in the fetus/infant rather than the mother, which would reduce specificity of the exposure. We thus conducted a deterministic bias analysis to examine how much misclassification (reduced specificity) would be required to nullify results. Non-differential reductions in specificity would be unlikely to attenuate our estimates, thus we assumed only reduced specificity amongst infants with adverse outcomes present. To evaluate this, we used mothers with Down Syndrome (Trisomy 21) as a test case from SOMI data (n=710 births; 53% of the chromosomal difference IDD group). Assuming 100% sensitivity for Down Syndrome exposure and 100% sensitivity among births without adverse outcomes, we identified the level of reduced exposure specificity among births with adverse outcomes needed to attenuate risk ratio estimates to the null. Our estimates were robust to small reductions in specificity across all adverse outcomes, with differential specificity needing to fall below 95% (NICU), 97% (PTB), 97% (very PTB), and 99% (SGA) before estimates were attenuated to the null (data not shown).

Programs

Probabilistic sensitivity analyses were completed using the R package, ‘episensr’.[2] Deterministic bias analyses were completed using methods described previously.[3]

© 2026 Psaras C et al. *JAMA Network Open.*

eReferences.

1. Dodds L, Spencer A, Shea S, et al. Validity of autism diagnoses using administrative health data. Chronic Diseases in Canada 2009;29(3):102-107.
2. Haine D. *The episensr package: basic sensitivity analysis of epidemiological results*. R package version 2.1.0; 2025. doi:10.32614/CRAN.package.episensr
3. Fox MP, MacLehose R, Lash TL. Applying Quantitative Bias Analysis to Epidemiologic Data. Springer. 2021.

© 2026 Psaras C et al. *JAMA Network Open.*

eTable 2. Demographic and Clinical Characteristics of IDD Sample, by IDD Subtype

	Autism Spectrum Disorder<br><br>N=458	Intellectual Disability<br><br>N=1,187	Cerebral Palsy<br><br>N=1,019	Chromosomal Differences<br><br>N=1,350	Other IDDs<br><br>N=600
Maternal Age at Birth, mean (SD)	26 (6)	27 (6)	29 (6)	32 (7)	29 (6)
Race/Ethnicity, n (%)					
Asian	16 (3%)	45 (4%)	40 (4%)	133 (10%)	33 (6%)
Hispanic	122 (27%)	542 (46%)	379 (37%)	635 (47%)	228 (38%)
Non-Hispanic Black	29 (6%)	219 (18%)	114 (11%)	54 (4%)	28 (5%)
Non-Hispanic White	232 (51%)	271 (23%)	406 (40%)	448 (33%)	259 (43%)
Other**	59 (13%)	110 (9%)	80 (8%)	80 (6%)	52 (9%)
Year of Birth, mean (SD)	2016 (3)	2014 (4)	2014 (4)	2015 (4)	2014 (4)
Maternal Education at Birth, n (%)					
<12 years	78 (17%)	346 (29%)	118 (12%)	301 (19%)	81 (14%)
>=12 years	341 (74%)	736 (62%)	840 (82%)	1,182 (75%)	480 (80%)
Missing	39 (9%)	105 (9%)	61 (6%)	88 (6%)	39 (6%)
WIC status, n (%)					
No WIC	169 (37%)	242 (20%)	397 (39%)	736 (55%)	317 (53%)
WIC	279 (61%)	916 (77%)	612 (60%)	595 (44%)	281 (47%)
Missing	<11 (<2%)	29 (2%)	<11 (<2%)	19 (1%)	<11 (<2%)
Payer, n (%)					
Private	149 (33%)	212 (18%)	394 (39%)	783 (58%)	328 (55%)
Public	307 (67%)	968 (82%)	620 (61%)	552 (41%)	272 (45%)
Other	<11 (<1%)	< 11(<1%)	<11 (<2%)	15 (1%)	<11 (<2%)
Rurality (see notes), median (IQR)	1 (1-3)	1 (1-3)	1 (1-3)	1 (1-3)	1 (1-3)
Adequate Prenatal Care (Dichotomized Kotelchuck Index), n (%)					
Inadequate or Intermediate	152 (33%)	475 (40%)	249 (24%)	273 (20%)	123 (20%)
Adequate or Adequate+	278 (61%)	621 (52%)	726 (71%)	1,009 (75%)	451 (75%)
Missing	28 (6%)	91 (8%)	44 (4%)	68 (5%)	26 (4%)
Body Mass Index (Dichotomized), n (%)					
Underweight or Normal	178 (39%)	413 (35%)	495 (49%)	578 (43%)	296 (49%)
Overweight or Obese	259 (57%)	656 (55%)	469 (46%)	715 (53%)	284 (47%)

© 2026 Psaras C et al. *JAMA Network Open.*

Missing	21 (5%)	118 (10%)	55 (5%)	57 (4%)	20 (3%)
Preexisting Hypertension, n (%)	31 (7%)	79 (7%)	63 (6%)	68 (5%)	50 (8%)
Preexisting Diabetes, n (%)	27 (6%)	80 (7%)	22 (2%)	30 (2%)	11 (2%)
Tobacco Use, n (%)	27 (6%)	57 (5%)	21 (2%)	11 (1%)	<11 (<2%)
Anxiety or Depression Diagnosis, n (%)	155 (34%)	271 (23%)	123 (12%)	127 (9%)	70 (12%)
Bipolar Disorder, n (%)	84 (18%)	185 (16%)	26 (3%)	<11 (<1%)	14 (2%)
Schizophrenia, n (%)	36 (8%)	146 (12%)	<11 (<2%)	<11 (<1%)	<11 (<2%)
Bipolar Disorder or Schizophrenia, n (%)	95 (21%)	252 (21%)	28 (3%)	<11 (1%)	17 (3%)
Epilepsy, n (%)	39 (9%)	142 (12%)	129 (13%)	16 (1%)	22 (4%)

Columns are not mutually exclusive; *=Matched 30:1 on maternal age (<=35 years old and >35 years old) and year of delivery; NICU= neonatal intensive care unit; WIC= USDA's Special Supplemental Nutrition Program for Women, Infants, and Children, BMI= body mass index; Rurality= Federal Information Processing Standards (FIPS) urban/rural county code continuum, 1 (most urban) to 6 (most rural); **= Other IDD composed of fetal alcohol syndrome, tuberous sclerosis, and various congenital malformations noted in Appendix Table S1

© 2026 Psaras C et al. *JAMA Network Open.*

eResults. Quantitative Bias Analyses Results

In the deterministic bias analysis (Table S3), we identified the reduced maternal IDD sensitivity among births without the adverse infant outcomes (differential misclassification of the exposure) required to attenuate aRR estimates to the null. The results varied by IDD subtype and outcome. For NICU admission, CP required the highest sensitivity (58%) while chromosomal differences required the lowest (29%). For SGA, ASD required the highest sensitivity (69%) while intellectual disability required the lowest (52%). For PTB, ASD required the highest sensitivity (48%) while ID required the lowest (35%). For very PTB, ASD required the highest sensitivity (50%) while other IDDs required the lowest (21%).

© 2026 Psaras C et al. *JAMA Network Open.*

eTable 3. Exposure Sensitivity Among Non-Cases Needed to Attenuate Risk Ratio Estimates to 1.0, by IDD Type and Outcome

IDD Type	NICU Admission	SGA	Very PTB	PTB
Any IDD	0.34	0.62	0.32	0.40
ASD	0.37	0.69	0.50	0.48
ID	0.32	0.52	0.30	0.35
CP	0.58	0.66	0.37	0.40
Chromosomal Differences	0.29	0.64	0.45	0.45
Other IDD	0.32	0.56	0.21	0.36

Misclassification allowed only among non-cases. Sensitivity of exposure classification in births with outcome assumed to be 100%. Specificity assumed to be 100%; RR= Risk Ratio; IDD=Intellectual and developmental disability; ASD= Autism Spectrum Disorders; ID=Intellectual Disability; CP=Cerebral Palsy; NICU=Neonatal intensive care unit admission; SGA=Small for gestational age; PTB=Preterm birth

© 2026 Psaras C et al. *JAMA Network Open.*

eTable 4. Results of Varying Sensitivity of Exposure Classification Between 7%-17%, Assuming Specificity is 100% and Misclassification of Exposure is Nondifferential

IDD Type	NICU Admission		SGA		Very PTB		PTB	
	Observed RR	Median Simulated RR (2.5th,97.5th)	Observed RR	Median Simulated RR (2.5th,97.5th)	Observed RR	Median Simulated RR (2.5th,97.5th)	Observed RR	Median Simulated RR (2.5th,97.5th)
Any IDD	2.76	5.90 (4.16, 62.88)	1.56	1.91 (1.71, 2.69)	3.20	8.41 (5.3, 115.88)	2.34	4.14 (3.15, 44.25)
Autism Spectrum Disorder (ASD)	2.59	5.17 (3.35, 49.16)	1.41	1.66 (1.35, 2.14)	2.04	3.3 (1.69, 13.29)	2.00	3.01 (2.2, 7.99)
Cerebral Palsy (CP)	1.71	2.27 (1.8, 3.59)	1.46	1.74 (1.48, 2.29)	2.82	6.78 (3.54, 87.96)	2.33	4.01 (2.99, 35.68)
Intellectual Disability (ID)	2.95	6.60 (4.52, 68.67)	1.80	2.40 (2.02, 4.25)	3.50	10.76 (5.47, 137.61)	2.64	5.42 (3.76, 47.06)
Chromosomal Differences	3.20	8.38 (5.43, 95.02)	1.51	1.84 (1.58, 2.45)	2.26	3.93 (2.62, 28.89)	2.10	3.31 (2.54, 11.52)
Other IDD**	2.99	6.84 (4.53, 80.04)	1.70	2.24 (1.81, 3.68)	5.06	45.4 (17.23, 460.82)	2.56	4.87 (3.46, 61.98)

IDD= Intellectual and developmental disability; NICU=Neonatal intensive care unit admission; SGA=Small for gestational age; PTB=Preterm birth; Observed RR is estimate from the 30:1 matched dataset, matched on year of delivery and maternal age at birth; Median (2.5th, 97.5th) estimates are from 1,000 simulations using the matched data and assuming sensitivity can vary uniformly between 0.07 and 0.17 and that specificity is constant at 1.0.; **= Other IDD composed of fetal alcohol syndrome, tuberous sclerosis, and various congenital malformations noted in Appendix Table S1

© 2026 Psaras C et al. *JAMA Network Open.*

eTable 5. Risk Ratios for Total, Direct and Indirect Effects and Proportion Mediated, by IDD/IDD Subtype, Outcome, and Mediator – NICU Admission

Outcome	Exposure	Mediator	Total Effect (95% CI)	Direct Effect (95% CI)	Indirect Effect (95% CI)	Proportion Mediated (95% CI)
NICU Admission	Any IDD	Adequate Prenatal Care	2.87 (2.60, 3.11)	2.88 (2.61, 3.12)	1.00 (1.00, 1.00)	
NICU Admission	Any IDD	Bipolar/Schizophrenia	2.82 (2.58, 3.09)	2.71 (2.47, 2.99)	1.04 (1.01, 1.06)	5.8% (2.2%, 9.0%)
NICU Admission	Any IDD	Depression/Anxiety	2.81 (2.57, 3.09)	2.67 (2.44, 2.94)	1.05 (1.04, 1.07)	7.6% (5.4%, 10.2%)
NICU Admission	Any IDD	Epilepsy	2.81 (2.55, 3.06)	2.75 (2.49, 3.00)	1.02 (1.00, 1.04)	3.1% (0.5%, 6.2%)
NICU Admission	Any IDD	Pre-pregnancy BMI	2.81 (2.55, 3.09)	2.78 (2.53, 3.06)	1.01 (1.00, 1.01)	1.4% (0.7%, 2.0%)
NICU Admission	Any IDD	Preexisting Diabetes	2.83 (2.58, 3.11)	2.73 (2.49, 2.99)	1.04 (1.03, 1.05)	5.7% (3.9%, 7.9%)
NICU Admission	Any IDD	Preexisting Hypertension	2.83 (2.59, 3.09)	2.65 (2.42, 2.89)	1.07 (1.05, 1.09)	10.1% (7.7%, 12.8%)
NICU Admission	Any IDD	Preterm Birth <37 weeks	2.86 (2.59, 3.11)	1.89 (1.74, 2.05)	1.51 (1.43, 1.59)	52.1% (46.9%, 56.7%)
NICU Admission	Any IDD	Tobacco Use	2.87 (2.61, 3.12)	2.82 (2.58, 3.07)	1.02 (1.01, 1.03)	2.5% (0.9%, 4.4%)
NICU Admission	Any IDD	Joint	3.24 (2.83, 3.65)	1.99 (1.74, 2.26)	1.63 (1.53, 1.73)	56.0% (50.1%, 61.8%)
NICU Admission	Maternal Autism	Adequate Prenatal Care	1.63 (0.94, 2.47)	1.65 (0.95, 2.48)	0.99 (0.96, 1.00)	
NICU Admission	Maternal Autism	Bipolar/Schizophrenia	1.56 (0.85, 2.60)	1.41 (0.71, 2.39)	1.10 (0.91, 1.39)	26.2% (-131.4%, 189.5%)
NICU Admission	Maternal Autism	Depression/Anxiety	1.74 (0.94, 2.80)	1.51 (0.78, 2.47)	1.15 (1.00, 1.32)	30.7% (-58.0%, 219.7%)
NICU Admission	Maternal Autism	Pre-pregnancy BMI	1.53 (0.85, 2.34)	1.49 (0.84, 2.30)	1.03 (1.00, 1.05)	7.5% (-21.9%, 35.1%)
NICU Admission	Maternal Autism	Preexisting Hypertension	1.77 (0.99, 2.67)	1.58 (0.86, 2.40)	1.12 (1.03, 1.27)	25.1% (-13.0%, 104.6%)
NICU Admission	Maternal Autism	Preterm Birth <37 weeks	1.74 (0.99, 2.66)	1.26 (0.69, 2.01)	1.38 (1.05, 1.76)	65.0% (-44.6%, 252.4%)
NICU Admission	Maternal Autism	Tobacco Use	1.64 (0.99, 2.51)	1.47 (0.88, 2.25)	1.12 (1.02, 1.26)	26.7% (-15.8%, 105.1%)

© 2026 Psaras C et al. *JAMA Network Open.*

eTable 5. Risk Ratios for Total, Direct and Indirect Effects and Proportion Mediated, by IDD/IDD Subtype, Outcome, and Mediator – NICU Admission

Outcome	Exposure	Mediator	Total Effect (95% CI)	Direct Effect (95% CI)	Indirect Effect (95% CI)	Proportion Mediated (95% CI)
NICU Admission	Maternal Autism	Joint	1.56 (0.79, 2.81)	0.86 (0.43, 1.58)	1.81 (1.20, 2.84)	
NICU Admission	Maternal Cerebral Palsy	Adequate Prenatal Care	1.86 (1.35, 2.40)	1.86 (1.35, 2.40)	1.00 (0.99, 1.01)	
NICU Admission	Maternal Cerebral Palsy	Depression/Anxiety	1.90 (1.38, 2.47)	1.81 (1.32, 2.34)	1.05 (1.02, 1.09)	10.1% (3.8%, 20.4%)
NICU Admission	Maternal Cerebral Palsy	Epilepsy	1.85 (1.38, 2.40)	1.67 (1.22, 2.21)	1.11 (1.01, 1.27)	21.2% (1.8%, 55.2%)
NICU Admission	Maternal Cerebral Palsy	Pre-pregnancy BMI	1.73 (1.31, 2.22)	1.74 (1.32, 2.23)	1.00 (0.99, 1.00)	
NICU Admission	Maternal Cerebral Palsy	Preexisting Hypertension	1.95 (1.50, 2.54)	1.78 (1.33, 2.32)	1.09 (1.06, 1.16)	17.6% (10.1%, 34.9%)
NICU Admission	Maternal Cerebral Palsy	Preterm Birth <37 weeks	1.85 (1.34, 2.49)	1.20 (0.88, 1.52)	1.54 (1.38, 1.75)	76.0% (59.9%, 133.9%)
NICU Admission	Maternal Cerebral Palsy	Joint	1.95 (1.46, 2.62)	1.04 (0.70, 1.42)	1.88 (1.58, 2.41)	96.1% (70.0%, 161.0%)
NICU Admission	Maternal Chromosomal Difference	Adequate Prenatal Care	3.18 (2.74, 3.63)	3.17 (2.73, 3.62)	1.00 (1.00, 1.01)	0.5% (-0.0%, 1.1%)
NICU Admission	Maternal Chromosomal Difference	Depression/Anxiety	3.06 (2.62, 3.54)	2.91 (2.49, 3.36)	1.05 (1.03, 1.08)	7.1% (4.3%, 10.7%)
NICU Admission	Maternal Chromosomal Difference	Pre-pregnancy BMI	3.15 (2.71, 3.64)	3.13 (2.69, 3.63)	1.01 (1.00, 1.01)	0.9% (0.2%, 2.0%)
NICU Admission	Maternal Chromosomal Difference	Preexisting Diabetes	3.06 (2.63, 3.50)	3.03 (2.61, 3.46)	1.01 (0.99, 1.04)	1.6% (-1.1%, 5.2%)
NICU Admission	Maternal Chromosomal Difference	Preexisting Hypertension	3.05 (2.62, 3.46)	2.94 (2.50, 3.32)	1.04 (1.02, 1.07)	5.5% (2.2%, 9.7%)
NICU Admission	Maternal Chromosomal Difference	Preterm Birth <37 weeks	3.17 (2.71, 3.65)	2.23 (1.90, 2.56)	1.42 (1.30, 1.55)	43.2% (34.7%, 51.4%)
NICU Admission	Maternal Chromosomal Difference	Joint	3.61 (2.96, 4.34)	2.54 (2.07, 3.07)	1.42 (1.30, 1.57)	40.9% (32.5%, 50.0%)
NICU Admission	Maternal Intellectual Disability	Adequate Prenatal Care	3.05 (2.44, 3.67)	3.07 (2.46, 3.69)	0.99 (0.98, 1.01)	
NICU Admission	Maternal Intellectual Disability	Bipolar/Schizophrenia	2.88 (2.33, 3.52)	2.78 (2.23, 3.45)	1.03 (0.95, 1.13)	5.1% (-7.5%, 18.1%)
NICU Admission	Maternal Intellectual Disability	Depression/Anxiety	2.96 (2.45, 3.58)	2.77 (2.26, 3.33)	1.07 (1.02, 1.13)	9.4% (2.6%, 17.5%)

© 2026 Psaras C et al. *JAMA Network Open.*

eTable 5. Risk Ratios for Total, Direct and Indirect Effects and Proportion Mediated, by IDD/IDD Subtype, Outcome, and Mediator – NICU Admission

Outcome	Exposure	Mediator	Total Effect (95% CI)	Direct Effect (95% CI)	Indirect Effect (95% CI)	Proportion Mediated (95% CI)
NICU Admission	Maternal Intellectual Disability	Epilepsy	2.91 (2.38, 3.49)	2.89 (2.33, 3.48)	1.01 (0.96, 1.07)	1.1% (-6.0%, 9.3%)
NICU Admission	Maternal Intellectual Disability	Pre-pregnancy BMI	3.06 (2.42, 3.69)	3.00 (2.38, 3.61)	1.02 (1.01, 1.03)	3.0% (1.0%, 5.0%)
NICU Admission	Maternal Intellectual Disability	Preexisting Diabetes	3.06 (2.50, 3.68)	2.73 (2.20, 3.30)	1.12 (1.06, 1.20)	16.0% (9.0%, 24.5%)
NICU Admission	Maternal Intellectual Disability	Preexisting Hypertension	2.98 (2.41, 3.56)	2.78 (2.24, 3.34)	1.07 (1.04, 1.11)	9.8% (5.4%, 15.2%)
NICU Admission	Maternal Intellectual Disability	Preterm Birth <37 weeks	2.97 (2.36, 3.49)	1.85 (1.53, 2.17)	1.60 (1.40, 1.75)	56.8% (45.2%, 65.4%)
NICU Admission	Maternal Intellectual Disability	Tobacco Use	2.91 (2.33, 3.52)	2.80 (2.25, 3.42)	1.04 (1.00, 1.08)	5.8% (0.5%, 11.7%)
NICU Admission	Maternal Intellectual Disability	Joint	3.42 (2.56, 4.22)	1.85 (1.33, 2.44)	1.85 (1.54, 2.16)	64.8% (49.9%, 79.8%)
NICU Admission	Other Maternal IDD	Adequate Prenatal Care	3.27 (2.56, 4.03)	3.24 (2.53, 4.00)	1.01 (1.00, 1.02)	1.5% (-0.0%, 3.2%)
NICU Admission	Other Maternal IDD	Bipolar/Schizophren ia	3.16 (2.48, 3.96)	3.13 (2.44, 3.92)	1.01 (0.99, 1.04)	1.4% (-1.3%, 5.8%)
NICU Admission	Other Maternal IDD	Depression/Anxiety	3.33 (2.57, 4.18)	3.20 (2.49, 4.02)	1.04 (1.01, 1.08)	5.3% (1.9%, 11.1%)
NICU Admission	Other Maternal IDD	Pre-pregnancy BMI	3.24 (2.53, 4.01)	3.24 (2.52, 4.00)	1.00 (0.99, 1.01)	0.1% (-0.9%, 1.1%)
NICU Admission	Other Maternal IDD	Preexisting Hypertension	3.09 (2.44, 3.86)	2.85 (2.21, 3.63)	1.08 (1.04, 1.16)	11.3% (5.4%, 20.6%)
NICU Admission	Other Maternal IDD	Preterm Birth <37 weeks	3.26 (2.59, 4.12)	1.87 (1.52, 2.25)	1.74 (1.52, 2.03)	61.5% (51.2%, 71.5%)
NICU Admission	Other Maternal IDD	Tobacco Use	3.18 (2.52, 3.95)	3.18 (2.52, 3.96)	1.00 (0.99, 1.01)	0.1% (-1.3%, 1.5%)
NICU Admission	Other Maternal IDD	Joint	3.37 (2.48, 4.59)	1.88 (1.40, 2.53)	1.79 (1.59, 2.16)	62.7% (51.8%, 77.0%)

Formulas*:

Direct Effect: The effect on NICU admission, on the ratio scale, of changing exposure to present vs absent, assuming the mediator the mediator is set to the value it would have taken if the exposure

© 2026 Psaras C et al. *JAMA Network Open.*

was present. In other words, a direct effect is the effect of exposure, in this case IDD or one of its subtypes, that does not go through the mediator(s) of interest.

Indirect Effect: The effect on NICU admission, on the ratio scale, of changing the mediator from what it would have been if the exposure was present to what it would have been if the exposure was absent, holding the value of the exposure to present. In other words, an indirect effect is the effect of the exposure that goes through the mediator(s) of interest.

Proportion mediated: (Direct Effect∗(Indirect Effect−1))/(Total Effect−1)

*For further details see *CMAverse* R package documentation.

Joint mediator model for NICU admission & Any IDD includes: Preterm Birth <37 weeks, Pre-pregnancy BMI, Preexisting Diabetes, Preexisting Hypertension, Depression/Anxiety, Epilepsy, Bipolar/Schizophrenia, Tobacco Use, Adequate Prenatal Care

Joint mediator model for NICU admission & Maternal Autism includes: Preterm Birth <37 weeks, Pre-pregnancy BMI, Preexisting Hypertension, Depression/Anxiety, Bipolar/Schizophrenia, Tobacco Use, Adequate Prenatal Care

Joint mediator model for NICU admission & Maternal Cerebral Palsy includes: Preterm Birth <37 weeks, Pre-pregnancy BMI, Preexisting Hypertension, Depression/Anxiety, Epilepsy, Adequate Prenatal Care

Joint mediator model for NICU admission & Maternal Chromosomal Difference includes: Preterm Birth <37 weeks, Pre-pregnancy BMI, Preexisting Diabetes, Preexisting Hypertension, Depression/Anxiety, Adequate Prenatal Care

Joint mediator model for NICU admission & Maternal Intellectual Disability includes: Preterm Birth <37 weeks, Pre-pregnancy BMI, Preexisting Diabetes, Preexisting Hypertension, Depression/Anxiety, Epilepsy, Bipolar/Schizophrenia, Tobacco Use, Adequate Prenatal Care

Joint mediator model for NICU admission & Other Maternal IDD includes: Preterm Birth <37 weeks, Pre-pregnancy BMI, Preexisting Hypertension, Depression/Anxiety, Bipolar/Schizophrenia, Adequate Prenatal Care

*Sample size insufficient for estimates of: Maternal Chromosomal Difference × Bipolar/Schizophrenia; Maternal Chromosomal Difference × Epilepsy; Maternal Chromosomal Difference × Tobacco Use; Maternal Autism × Epilepsy; Maternal Autism × Preexisting Diabetes; Maternal Cerebral Palsy × Bipolar/Schizophrenia; Maternal Cerebral Palsy × Preexisting Diabetes; Maternal Cerebral Palsy × Tobacco Use; Other Maternal IDD × Epilepsy; Other Maternal IDD × Preexisting Diabetes.

© 2026 Psaras C et al. *JAMA Network Open.*

eTable 6. Risk Ratios for Total, Direct and Indirect Effects and Proportion Mediated, by IDD/IDD Subtype, Outcome, and Mediator – Preterm Birth <32 Weeks

Outcome	Exposure	Mediator	Total Effect (95% CI)	Direct Effect (95% CI)	Indirect Effect (95% CI)	Proportion Mediated (95% CI)
Preterm Birth <32 Weeks	Any IDD	Adequate Prenatal Care	2.78 (2.12, 3.54)	2.79 (2.14, 3.56)	0.99 (0.99, 1.00)	
Preterm Birth <32 Weeks	Any IDD	Bipolar/Schizophrenia	2.65 (2.01, 3.33)	2.57 (1.91, 3.30)	1.03 (0.98, 1.10)	4.7% (-3.2%, 15.5%)
Preterm Birth <32 Weeks	Any IDD	Depression/Anxiety	2.99 (2.26, 3.80)	2.79 (2.10, 3.51)	1.07 (1.03, 1.12)	10.3% (4.8%, 16.5%)
Preterm Birth <32 Weeks	Any IDD	Epilepsy	2.79 (2.14, 3.52)	2.60 (1.98, 3.31)	1.07 (1.01, 1.15)	10.5% (1.6%, 21.2%)
Preterm Birth <32 Weeks	Any IDD	Pre-pregnancy BMI	2.73 (2.08, 3.59)	2.69 (2.04, 3.53)	1.02 (1.01, 1.03)	2.6% (1.3%, 4.1%)
Preterm Birth <32 Weeks	Any IDD	Preexisting Hypertension	2.66 (2.01, 3.28)	2.36 (1.77, 2.96)	1.13 (1.08, 1.18)	18.1% (11.7%, 26.6%)
Preterm Birth <32 Weeks	Any IDD	Joint	2.77 (1.94, 3.66)	2.27 (1.56, 3.11)	1.22 (1.13, 1.34)	28.1% (17.6%, 45.7%)
Preterm Birth <32 Weeks	Maternal Cerebral Palsy	Pre-pregnancy BMI	3.19 (1.65, 5.23)	3.24 (1.68, 5.23)	0.99 (0.96, 1.00)	
Preterm Birth <32 Weeks	Maternal Cerebral Palsy	Preexisting Diabetes	3.61 (1.65, 6.11)	3.48 (1.61, 5.91)	1.04 (1.00, 1.10)	5.2% (0.0%, 15.1%)
Preterm Birth <32 Weeks	Maternal Cerebral Palsy	Joint	3.23 (1.61, 5.28)	3.29 (1.65, 5.31)	0.98 (0.96, 1.00)	
Preterm Birth <32 Weeks	Maternal Chromosomal Difference	Depression/Anxiety	2.42 (1.40, 3.63)	2.27 (1.32, 3.34)	1.07 (1.02, 1.12)	10.5% (3.1%, 23.3%)
Preterm Birth <32 Weeks	Maternal Chromosomal Difference	Pre-pregnancy BMI	2.29 (1.29, 3.39)	2.25 (1.28, 3.35)	1.01 (1.00, 1.03)	2.5% (0.3%, 6.3%)
Preterm Birth <32 Weeks	Maternal Chromosomal Difference	Preexisting Diabetes	2.36 (1.46, 3.54)	2.35 (1.47, 3.50)	1.00 (1.00, 1.02)	0.8% (-0.5%, 3.5%)
Preterm Birth <32 Weeks	Maternal Chromosomal Difference	Joint	2.35 (1.27, 3.63)	2.16 (1.17, 3.37)	1.08 (1.02, 1.15)	13.5% (3.9%, 35.9%)
Preterm Birth <32 Weeks	Maternal Intellectual Disability	Adequate Prenatal Care	2.86 (1.55, 4.69)	2.95 (1.62, 4.78)	0.97 (0.93, 1.01)	

© 2026 Psaras C et al. *JAMA Network Open.*

eTable 6. Risk Ratios for Total, Direct and Indirect Effects and Proportion Mediated, by IDD/IDD Subtype, Outcome, and Mediator – Preterm Birth <32 Weeks

Outcome	Exposure	Mediator	Total Effect (95% CI)	Direct Effect (95% CI)	Indirect Effect (95% CI)	Proportion Mediated (95% CI)
Preterm Birth <32 Weeks	Maternal Intellectual Disability	Depression/Anxiety	3.14 (1.60, 5.04)	3.07 (1.62, 4.70)	1.02 (0.91, 1.16)	3.1% (-18.4%, 18.5%)
Preterm Birth <32 Weeks	Maternal Intellectual Disability	Pre-pregnancy BMI	2.58 (1.37, 4.07)	2.52 (1.34, 4.00)	1.02 (0.99, 1.06)	3.7% (-2.0%, 12.3%)
Preterm Birth <32 Weeks	Maternal Intellectual Disability	Joint	3.39 (1.46, 5.66)	3.26 (1.55, 5.06)	1.04 (0.90, 1.22)	5.2% (-23.6%, 24.0%)
Preterm Birth <32 Weeks	Other Maternal IDD	Pre-pregnancy BMI	8.08 (4.40, 12.62)	8.07 (4.38, 12.63)	1.00 (0.99, 1.02)	0.1% (-1.6%, 1.9%)
Preterm Birth <32 Weeks	Other Maternal IDD	Preexisting Diabetes	7.13 (4.02, 10.85)	6.98 (3.88, 10.67)	1.02 (0.99, 1.08)	2.4% (-1.4%, 8.6%)
Preterm Birth <32 Weeks	Other Maternal IDD	Joint	8.54 (4.37, 13.96)	8.53 (4.36, 13.91)	1.00 (0.98, 1.02)	0.1% (-1.7%, 2.0%)

Formulas*:

Direct Effect: The effect on PTB, on the ratio scale, of changing exposure to present vs absent, assuming the mediator the mediator is set to the value it would have taken if the exposure was present. In other words, a direct effect is the effect of exposure, in this case IDD or one of its subtypes, that does not go through the mediator(s) of interest.

Indirect Effect: The effect on PTB, on the ratio scale, of changing the mediator from what it would have been if the exposure was present to what it would have been if the exposure was absent, holding the value of the exposure to present. In other words, an indirect effect is the effect of the exposure that goes through the mediator(s) of interest.

Proportion mediated: (Direct Effect∗(Indirect Effect−1))/(Total Effect−1)

*For further details see *CMAverse* R package documentation.

Joint mediator model for preterm birth <32 weeks & Any IDD includes: Adequate Prenatal Care, Pre-pregnancy BMI, Preexisting Hypertension, Depression/Anxiety, Epilepsy, Bipolar/Schizophrenia

Joint mediator model for preterm birth <32 weeks & Maternal Cerebral Palsy includes: Pre-pregnancy BMI

Joint mediator model for preterm birth <32 weeks & Maternal Chromosomal Difference includes: Pre-pregnancy BMI, Depression/Anxiety

© 2026 Psaras C et al. *JAMA Network Open.*

Joint mediator model for preterm birth <32 weeks & Maternal Intellectual Disability includes: Adequate Prenatal Care, Pre-pregnancy BMI, Depression/Anxiety

Joint mediator model for preterm birth <32 weeks & Other Maternal IDD includes: Pre-pregnancy BMI

*Sample size insufficient for estimates of: Maternal Chromosomal Difference × Bipolar/Schizophrenia; Maternal Chromosomal Difference × Epilepsy; Maternal Chromosomal Difference × Adequate Prenatal Care; Maternal Chromosomal Difference × Preexisting Hypertension; Maternal Chromosomal Difference × Tobacco Use; Any IDD × Preexisting Diabetes; Any IDD × Tobacco Use; Maternal Autism × Pre-pregnancy BMI; Maternal Autism × Bipolar/Schizophrenia; Maternal Autism × Epilepsy; Maternal Autism × Adequate Prenatal Care; Maternal Autism × Depression/Anxiety; Maternal Autism × Preexisting Diabetes; Maternal Autism × Preexisting Hypertension; Maternal Autism × Tobacco Use; Maternal Cerebral Palsy × Bipolar/Schizophrenia; Maternal Cerebral Palsy × Epilepsy; Maternal Cerebral Palsy × Adequate Prenatal Care; Maternal Cerebral Palsy × Depression/Anxiety; Maternal Cerebral Palsy × Preexisting Hypertension; Maternal Cerebral Palsy × Tobacco Use; Maternal Intellectual Disability × Bipolar/Schizophrenia; Maternal Intellectual Disability × Epilepsy; Maternal Intellectual Disability × Preexisting Diabetes; Maternal Intellectual Disability × Preexisting Hypertension; Maternal Intellectual Disability × Tobacco Use; Other Maternal IDD × Bipolar/Schizophrenia; Other Maternal IDD × Epilepsy; Other Maternal IDD × Adequate Prenatal Care; Other Maternal IDD × Depression/Anxiety; Other Maternal IDD × Preexisting Hypertension; Other Maternal IDD × Tobacco Use.

© 2026 Psaras C et al. *JAMA Network Open.*

eTable 7. Risk Ratios for Total, Direct and Indirect Effects and Proportion Mediated, by IDD/IDD Subtype, Outcome, and Mediator – Preterm Birth <37 Weeks

Outcome	Exposure	Mediator	Total Effect (95% CI)	Direct Effect (95% CI)	Indirect Effect (95% CI)	Proportion Mediated (95% CI)
Preterm Birth <37 weeks	Any IDD	Adequate Prenatal Care	2.32 (2.12, 2.54)	2.34 (2.13, 2.55)	0.99 (0.99, 1.00)	
Preterm Birth <37 weeks	Any IDD	Bipolar/Schizophrenia	2.28 (2.08, 2.48)	2.22 (2.03, 2.42)	1.03 (1.01, 1.05)	4.6% (1.4%, 7.9%)
Preterm Birth <37 weeks	Any IDD	Depression/Anxiety	2.29 (2.10, 2.50)	2.21 (2.03, 2.41)	1.04 (1.02, 1.05)	6.2% (3.9%, 8.5%)
Preterm Birth <37 weeks	Any IDD	Epilepsy	2.28 (2.09, 2.48)	2.23 (2.04, 2.43)	1.02 (1.01, 1.04)	3.9% (1.0%, 7.2%)
Preterm Birth <37 weeks	Any IDD	Pre-pregnancy BMI	2.31 (2.08, 2.52)	2.29 (2.06, 2.50)	1.01 (1.00, 1.01)	1.1% (0.6%, 1.7%)
Preterm Birth <37 weeks	Any IDD	Preexisting Diabetes	2.33 (2.13, 2.54)	2.24 (2.05, 2.43)	1.04 (1.03, 1.05)	6.8% (4.6%, 9.2%)
Preterm Birth <37 weeks	Any IDD	Preexisting Hypertension	2.31 (2.09, 2.50)	2.15 (1.95, 2.33)	1.07 (1.06, 1.09)	12.1% (9.3%, 15.2%)
Preterm Birth <37 weeks	Any IDD	Tobacco Use	2.32 (2.12, 2.52)	2.30 (2.10, 2.49)	1.01 (1.00, 1.02)	1.9% (0.5%, 3.6%)
Preterm Birth <37 weeks	Any IDD	Joint	2.55 (2.25, 2.89)	2.22 (1.96, 2.54)	1.15 (1.10, 1.20)	21.2% (15.3%, 28.0%)
Preterm Birth <37 weeks	Maternal Autism	Adequate Prenatal Care	1.94 (1.13, 2.99)	1.99 (1.18, 3.04)	0.98 (0.94, 1.00)	
Preterm Birth <37 weeks	Maternal Autism	Depression/Anxiety	1.89 (1.08, 2.83)	1.70 (0.93, 2.64)	1.11 (1.00, 1.26)	21.7% (-4.3%, 101.7%)
Preterm Birth <37 weeks	Maternal Autism	Pre-pregnancy BMI	1.74 (1.01, 2.70)	1.71 (1.00, 2.65)	1.01 (1.00, 1.04)	3.3% (-2.3%, 16.0%)
Preterm Birth <37 weeks	Maternal Autism	Preexisting Diabetes	2.05 (1.16, 3.10)	1.91 (1.08, 2.93)	1.08 (1.02, 1.15)	14.1% (2.8%, 43.0%)
Preterm Birth <37 weeks	Maternal Autism	Preexisting Hypertension	1.91 (1.11, 2.93)	1.64 (0.99, 2.46)	1.16 (1.04, 1.32)	28.9% (6.6%, 81.3%)
Preterm Birth <37 weeks	Maternal Autism	Joint	1.42 (0.75, 2.77)	1.17 (0.61, 2.29)	1.21 (1.01, 1.44)	59.5% (-420.3%, 517.3%)
Preterm Birth <37 weeks	Maternal Cerebral Palsy	Adequate Prenatal Care	2.33 (1.88, 2.83)	2.33 (1.89, 2.81)	1.00 (0.99, 1.01)	
Preterm Birth <37 weeks	Maternal Cerebral Palsy	Bipolar/Schizophrenia	2.37 (1.91, 2.87)	2.35 (1.89, 2.87)	1.01 (0.99, 1.03)	1.2% (-1.8%, 5.9%)

© 2026 Psaras C et al. *JAMA Network Open.*

eTable 7. Risk Ratios for Total, Direct and Indirect Effects and Proportion Mediated, by IDD/IDD Subtype, Outcome, and Mediator – Preterm Birth <37 Weeks

Outcome	Exposure	Mediator	Total Effect (95% CI)	Direct Effect (95% CI)	Indirect Effect (95% CI)	Proportion Mediated (95% CI)
Preterm Birth <37 weeks	Maternal Cerebral Palsy	Depression/Anxiety	2.38 (1.91, 2.85)	2.32 (1.85, 2.79)	1.03 (1.01, 1.05)	4.6% (1.0%, 8.9%)
Preterm Birth <37 weeks	Maternal Cerebral Palsy	Epilepsy	2.38 (1.98, 2.88)	2.30 (1.85, 2.82)	1.04 (0.98, 1.10)	6.0% (-2.8%, 17.1%)
Preterm Birth <37 weeks	Maternal Cerebral Palsy	Pre-pregnancy BMI	2.21 (1.79, 2.63)	2.21 (1.80, 2.64)	1.00 (0.99, 1.00)	
Preterm Birth <37 weeks	Maternal Cerebral Palsy	Preexisting Hypertension	2.46 (1.98, 2.97)	2.24 (1.79, 2.70)	1.10 (1.05, 1.15)	15.4% (8.3%, 23.3%)
Preterm Birth <37 weeks	Maternal Cerebral Palsy	Tobacco Use	2.43 (1.95, 2.92)	2.36 (1.90, 2.84)	1.03 (1.00, 1.06)	4.8% (0.8%, 10.1%)
Preterm Birth <37 weeks	Maternal Cerebral Palsy	Joint	2.41 (1.91, 3.11)	2.12 (1.63, 2.74)	1.14 (1.07, 1.26)	20.6% (10.3%, 36.5%)
Preterm Birth <37 weeks	Maternal Chromosomal Difference	Adequate Prenatal Care	2.10 (1.81, 2.43)	2.09 (1.80, 2.40)	1.01 (1.00, 1.01)	1.3% (0.1%, 2.8%)
Preterm Birth <37 weeks	Maternal Chromosomal Difference	Depression/Anxiety	2.08 (1.76, 2.41)	2.03 (1.70, 2.35)	1.03 (1.01, 1.05)	5.1% (2.7%, 9.1%)
Preterm Birth <37 weeks	Maternal Chromosomal Difference	Pre-pregnancy BMI	2.10 (1.76, 2.48)	2.09 (1.75, 2.47)	1.01 (1.00, 1.01)	1.3% (0.3%, 2.6%)
Preterm Birth <37 weeks	Maternal Chromosomal Difference	Preexisting Diabetes	2.07 (1.77, 2.41)	2.05 (1.75, 2.38)	1.01 (1.00, 1.03)	2.1% (-0.9%, 5.0%)
Preterm Birth <37 weeks	Maternal Chromosomal Difference	Preexisting Hypertension	2.06 (1.76, 2.38)	1.96 (1.67, 2.26)	1.05 (1.02, 1.08)	9.8% (3.3%, 15.5%)
Preterm Birth <37 weeks	Maternal Chromosomal Difference	Joint	2.32 (1.90, 2.79)	2.16 (1.74, 2.62)	1.07 (1.04, 1.11)	11.9% (7.0%, 18.6%)
Preterm Birth <37 weeks	Maternal Intellectual Disability	Adequate Prenatal Care	2.62 (2.15, 3.08)	2.72 (2.23, 3.20)	0.96 (0.95, 0.98)	
Preterm Birth <37 weeks	Maternal Intellectual Disability	Bipolar/Schizophrenia	2.53 (2.06, 3.02)	2.53 (2.05, 3.03)	1.00 (0.93, 1.08)	
Preterm Birth <37 weeks	Maternal Intellectual Disability	Depression/Anxiety	2.59 (2.12, 3.09)	2.46 (2.02, 2.95)	1.05 (1.01, 1.10)	8.3% (0.9%, 15.4%)

© 2026 Psaras C et al. *JAMA Network Open.*

eTable 7. Risk Ratios for Total, Direct and Indirect Effects and Proportion Mediated, by IDD/IDD Subtype, Outcome, and Mediator – Preterm Birth <37 Weeks

Outcome	Exposure	Mediator	Total Effect (95% CI)	Direct Effect (95% CI)	Indirect Effect (95% CI)	Proportion Mediated (95% CI)
Preterm Birth <37 weeks	Maternal Intellectual Disability	Epilepsy	2.52 (2.08, 3.01)	2.53 (2.07, 3.04)	1.00 (0.95, 1.04)	
Preterm Birth <37 weeks	Maternal Intellectual Disability	Pre-pregnancy BMI	2.60 (2.13, 3.14)	2.58 (2.11, 3.12)	1.01 (1.00, 1.02)	1.7% (0.0%, 3.4%)
Preterm Birth <37 weeks	Maternal Intellectual Disability	Preexisting Diabetes	2.61 (2.13, 3.11)	2.35 (1.92, 2.78)	1.11 (1.06, 1.17)	15.7% (9.2%, 23.7%)
Preterm Birth <37 weeks	Maternal Intellectual Disability	Preexisting Hypertension	2.52 (2.06, 3.00)	2.35 (1.91, 2.83)	1.07 (1.04, 1.11)	11.0% (6.5%, 17.2%)
Preterm Birth <37 weeks	Maternal Intellectual Disability	Tobacco Use	2.51 (2.03, 3.02)	2.42 (1.95, 2.92)	1.04 (1.00, 1.08)	5.7% (0.8%, 12.2%)
Preterm Birth <37 weeks	Maternal Intellectual Disability	Joint	2.82 (2.09, 3.73)	2.53 (1.84, 3.44)	1.12 (0.99, 1.26)	16.0% (-2.4%, 34.2%)
Preterm Birth <37 weeks	Other Maternal IDD	Adequate Prenatal Care	3.04 (2.45, 3.69)	3.01 (2.42, 3.65)	1.01 (1.00, 1.02)	1.4% (-0.0%, 3.0%)
Preterm Birth <37 weeks	Other Maternal IDD	Bipolar/Schizophrenia	2.96 (2.34, 3.62)	2.93 (2.30, 3.60)	1.01 (0.99, 1.05)	1.7% (-1.6%, 6.9%)
Preterm Birth <37 weeks	Other Maternal IDD	Depression/Anxiety	3.02 (2.38, 3.68)	2.92 (2.29, 3.57)	1.03 (1.01, 1.07)	5.0% (1.3%, 10.0%)
Preterm Birth <37 weeks	Other Maternal IDD	Pre-pregnancy BMI	3.24 (2.52, 4.00)	3.24 (2.52, 3.99)	1.00 (1.00, 1.01)	0.1% (-0.6%, 0.8%)
Preterm Birth <37 weeks	Other Maternal IDD	Preexisting Hypertension	2.90 (2.29, 3.57)	2.66 (2.08, 3.27)	1.09 (1.05, 1.15)	12.7% (6.8%, 20.4%)
Preterm Birth <37 weeks	Other Maternal IDD	Tobacco Use	2.91 (2.32, 3.57)	2.90 (2.32, 3.57)	1.00 (0.99, 1.02)	0.3% (-0.9%, 2.3%)
Preterm Birth <37 weeks	Other Maternal IDD	Joint	3.79 (2.76, 4.94)	3.40 (2.46, 4.44)	1.11 (1.06, 1.18)	14.0% (7.4%, 21.6%)

Formulas*:

Direct Effect: The effect on PTB, on the ratio scale, of changing exposure to present vs absent, assuming the mediator the mediator is set to the value it would have taken if the exposure was present. In other words, a direct effect is the effect of exposure, in this case IDD or one of its subtypes, that does not go through the mediator(s) of interest.

Indirect Effect: The effect on PTB, on the ratio scale, of changing the mediator from what it would have been if the exposure was present to what it would have been if the exposure was absent,

© 2026 Psaras C et al. *JAMA Network Open.*

holding the value of the exposure to present. In other words, an indirect effect is the effect of the exposure that goes through the mediator(s) of interest.

Proportion mediated: (Direct Effect∗(Indirect Effect−1))/(Total Effect−1)

*For further details see *CMAverse* R package documentation.

Joint mediator model for preterm birth <37 weeks & Any IDD includes: Adequate Prenatal Care, Pre-pregnancy BMI, Preexisting Diabetes, Preexisting Hypertension, Depression/Anxiety, Epilepsy, Bipolar/Schizophrenia, Tobacco Use

Joint mediator model for preterm birth <37 weeks & Maternal Autism includes: Adequate Prenatal Care, Pre-pregnancy BMI, Preexisting Diabetes, Preexisting Hypertension, Depression/Anxiety

Joint mediator model for preterm birth <37 weeks & Maternal Cerebral Palsy includes: Adequate Prenatal Care, Pre-pregnancy BMI, Preexisting Hypertension, Depression/Anxiety, Epilepsy, Bipolar/Schizophrenia, Tobacco Use

Joint mediator model for preterm birth <37 weeks & Maternal Chromosomal Difference includes: Adequate Prenatal Care, Pre-pregnancy BMI, Preexisting Diabetes, Preexisting Hypertension, Depression/Anxiety

Joint mediator model for preterm birth <37 weeks & Maternal Intellectual Disability includes: Adequate Prenatal Care, Pre-pregnancy BMI, Preexisting Diabetes, Preexisting Hypertension, Depression/Anxiety, Epilepsy, Bipolar/Schizophrenia, Tobacco Use

Joint mediator model for preterm birth <37 weeks & Other Maternal IDD includes: Adequate Prenatal Care, Pre-pregnancy BMI, Preexisting Hypertension, Depression/Anxiety, Bipolar/Schizophrenia

*Sample size insufficient for estimates of: Maternal Chromosomal Difference × Bipolar/Schizophrenia; Maternal Chromosomal Difference × Epilepsy; Maternal Chromosomal Difference × Tobacco Use; Maternal Autism × Bipolar/Schizophrenia; Maternal Autism × Epilepsy; Maternal Autism × Tobacco Use; Maternal Cerebral Palsy × Preexisting Diabetes; Other Maternal IDD × Epilepsy; Other Maternal IDD × Preexisting Diabetes.

© 2026 Psaras C et al. *JAMA Network Open.*

eTable 8. Risk Ratios for Total, Direct and Indirect Effects and Proportion Mediated, by IDD/IDD Subtype, Outcome, and Mediator – Small for Gestational Age Infant

Outcome	Exposure	Mediator	Total Effect (95% CI)	Direct Effect (95% CI)	Indirect Effect (95% CI)	Proportion Mediated (95% CI)
Small for Gestational Age	Any IDD	Adequate Prenatal Care	1.60 (1.46, 1.74)	1.60 (1.46, 1.74)	1.00 (1.00, 1.00)	0.1% (-0.1%, 0.4%)
Small for Gestational Age	Any IDD	Bipolar/Schizophrenia	1.52 (1.38, 1.66)	1.50 (1.36, 1.65)	1.01 (0.99, 1.03)	2.9% (-1.9%, 8.4%)
Small for Gestational Age	Any IDD	Depression/Anxiety	1.55 (1.41, 1.70)	1.55 (1.40, 1.70)	1.00 (0.99, 1.02)	1.1% (-2.1%, 4.3%)
Small for Gestational Age	Any IDD	Epilepsy	1.57 (1.44, 1.70)	1.55 (1.42, 1.68)	1.01 (0.99, 1.03)	2.5% (-1.4%, 7.0%)
Small for Gestational Age	Any IDD	Pre-pregnancy BMI	1.60 (1.44, 1.76)	1.62 (1.45, 1.79)	0.99 (0.98, 0.99)	
Small for Gestational Age	Any IDD	Preexisting Diabetes	1.56 (1.41, 1.72)	1.57 (1.42, 1.73)	1.00 (0.99, 1.00)	
Small for Gestational Age	Any IDD	Preexisting Hypertension	1.58 (1.43, 1.73)	1.55 (1.40, 1.69)	1.02 (1.02, 1.03)	6.1% (4.0%, 8.9%)
Small for Gestational Age	Any IDD	Preterm Birth <37 weeks	1.59 (1.44, 1.75)	1.55 (1.41, 1.70)	1.03 (1.02, 1.04)	7.0% (4.9%, 9.8%)
Small for Gestational Age	Any IDD	Tobacco Use	1.56 (1.43, 1.71)	1.56 (1.42, 1.71)	1.00 (1.00, 1.01)	0.9% (-0.8%, 2.8%)
Small for Gestational Age	Any IDD	Joint	1.62 (1.43, 1.83)	1.53 (1.34, 1.74)	1.06 (1.03, 1.09)	14.1% (6.7%, 23.7%)
Small for Gestational Age	Maternal Autism	Adequate Prenatal Care	1.32 (0.73, 2.01)	1.32 (0.74, 2.02)	0.99 (0.97, 1.01)	
Small for Gestational Age	Maternal Autism	Bipolar/Schizophrenia	1.22 (0.72, 1.86)	1.34 (0.77, 2.04)	0.92 (0.82, 1.01)	
Small for Gestational Age	Maternal Autism	Depression/Anxiety	1.21 (0.75, 1.87)	1.28 (0.76, 2.01)	0.95 (0.86, 1.03)	
Small for Gestational Age	Maternal Autism	Pre-pregnancy BMI	1.21 (0.67, 1.86)	1.24 (0.68, 1.93)	0.97 (0.94, 1.00)	
Small for Gestational Age	Maternal Autism	Preexisting Diabetes	1.32 (0.77, 1.99)	1.33 (0.77, 2.01)	0.99 (0.97, 1.01)	
Small for Gestational Age	Maternal Autism	Preterm Birth <37 weeks	1.37 (0.79, 2.12)	1.35 (0.78, 2.07)	1.01 (1.00, 1.05)	5.0% (-23.5%, 33.0%)
Small for Gestational Age	Maternal Autism	Joint	1.04 (0.57, 1.80)	1.31 (0.69, 2.25)	0.80 (0.66, 1.01)	

© 2026 Psaras C et al. *JAMA Network Open.*

eTable 8. Risk Ratios for Total, Direct and Indirect Effects and Proportion Mediated, by IDD/IDD Subtype, Outcome, and Mediator – Small for Gestational Age Infant

Outcome	Exposure	Mediator	Total Effect (95% CI)	Direct Effect (95% CI)	Indirect Effect (95% CI)	Proportion Mediated (95% CI)
Small for Gestational Age	Maternal Cerebral Palsy	Adequate Prenatal Care	1.43 (1.13, 1.75)	1.43 (1.13, 1.75)	1.00 (1.00, 1.00)	0.1% (-1.3%, 1.6%)
Small for Gestational Age	Maternal Cerebral Palsy	Bipolar/Schizophrenia	1.41 (1.16, 1.71)	1.40 (1.15, 1.69)	1.00 (0.99, 1.02)	1.4% (-2.5%, 8.2%)
Small for Gestational Age	Maternal Cerebral Palsy	Depression/Anxiety	1.36 (1.09, 1.67)	1.37 (1.10, 1.68)	0.99 (0.98, 1.01)	
Small for Gestational Age	Maternal Cerebral Palsy	Epilepsy	1.38 (1.09, 1.68)	1.36 (1.06, 1.66)	1.02 (0.97, 1.07)	6.0% (-15.2%, 36.7%)
Small for Gestational Age	Maternal Cerebral Palsy	Pre-pregnancy BMI	1.28 (1.03, 1.58)	1.27 (1.02, 1.56)	1.01 (1.00, 1.02)	4.3% (-1.9%, 21.0%)
Small for Gestational Age	Maternal Cerebral Palsy	Preexisting Hypertension	1.41 (1.12, 1.72)	1.34 (1.06, 1.63)	1.05 (1.03, 1.09)	16.4% (7.6%, 47.6%)
Small for Gestational Age	Maternal Cerebral Palsy	Preterm Birth <37 weeks	1.39 (1.08, 1.70)	1.38 (1.07, 1.69)	1.01 (1.00, 1.03)	4.2% (-1.6%, 17.1%)
Small for Gestational Age	Maternal Cerebral Palsy	Joint	1.41 (1.07, 1.80)	1.27 (0.97, 1.62)	1.11 (1.03, 1.20)	33.7% (9.6%, 115.7%)
Small for Gestational Age	Maternal Chromosomal Difference	Adequate Prenatal Care	1.50 (1.26, 1.76)	1.50 (1.26, 1.76)	1.00 (1.00, 1.00)	
Small for Gestational Age	Maternal Chromosomal Difference	Depression/Anxiety	1.47 (1.25, 1.72)	1.46 (1.24, 1.71)	1.00 (0.99, 1.02)	1.4% (-2.5%, 5.6%)
Small for Gestational Age	Maternal Chromosomal Difference	Epilepsy	1.47 (1.23, 1.72)	1.48 (1.23, 1.72)	1.00 (0.99, 1.01)	
Small for Gestational Age	Maternal Chromosomal Difference	Pre-pregnancy BMI	1.56 (1.30, 1.83)	1.57 (1.32, 1.86)	0.99 (0.98, 1.00)	
Small for Gestational Age	Maternal Chromosomal Difference	Preexisting Hypertension	1.50 (1.27, 1.77)	1.48 (1.26, 1.75)	1.01 (1.00, 1.02)	2.5% (0.7%, 6.3%)
Small for Gestational Age	Maternal Chromosomal Difference	Preterm Birth <37 weeks	1.51 (1.28, 1.78)	1.49 (1.25, 1.74)	1.02 (1.01, 1.03)	5.2% (2.0%, 10.4%)
Small for Gestational Age	Maternal Chromosomal Difference	Joint	1.69 (1.35, 2.06)	1.64 (1.31, 2.01)	1.03 (1.00, 1.05)	6.6% (0.3%, 13.4%)

© 2026 Psaras C et al. *JAMA Network Open.*

eTable 8. Risk Ratios for Total, Direct and Indirect Effects and Proportion Mediated, by IDD/IDD Subtype, Outcome, and Mediator – Small for Gestational Age Infant

Outcome	Exposure	Mediator	Total Effect (95% CI)	Direct Effect (95% CI)	Indirect Effect (95% CI)	Proportion Mediated (95% CI)
Small for Gestational Age	Maternal Intellectual Disability	Adequate Prenatal Care	1.80 (1.46, 2.19)	1.79 (1.46, 2.18)	1.00 (0.99, 1.01)	0.8% (-1.9%, 3.5%)
Small for Gestational Age	Maternal Intellectual Disability	Bipolar/Schizophrenia	1.64 (1.33, 1.96)	1.76 (1.39, 2.14)	0.93 (0.88, 1.00)	
Small for Gestational Age	Maternal Intellectual Disability	Depression/Anxiety	1.80 (1.46, 2.13)	1.75 (1.42, 2.09)	1.03 (0.99, 1.06)	5.6% (-2.3%, 14.4%)
Small for Gestational Age	Maternal Intellectual Disability	Epilepsy	1.76 (1.45, 2.11)	1.77 (1.43, 2.13)	1.00 (0.96, 1.04)	
Small for Gestational Age	Maternal Intellectual Disability	Pre-pregnancy BMI	1.78 (1.46, 2.14)	1.85 (1.52, 2.22)	0.96 (0.95, 0.98)	
Small for Gestational Age	Maternal Intellectual Disability	Preexisting Diabetes	1.79 (1.49, 2.15)	1.82 (1.50, 2.19)	0.98 (0.96, 1.00)	
Small for Gestational Age	Maternal Intellectual Disability	Preexisting Hypertension	1.80 (1.49, 2.16)	1.79 (1.47, 2.13)	1.01 (1.00, 1.02)	2.1% (-0.6%, 5.6%)
Small for Gestational Age	Maternal Intellectual Disability	Preterm Birth <37 weeks	1.78 (1.45, 2.13)	1.69 (1.37, 2.03)	1.05 (1.03, 1.08)	11.4% (6.6%, 18.8%)
Small for Gestational Age	Maternal Intellectual Disability	Tobacco Use	1.73 (1.45, 2.07)	1.74 (1.45, 2.09)	0.99 (0.98, 1.01)	
Small for Gestational Age	Maternal Intellectual Disability	Joint	1.64 (1.27, 2.10)	1.71 (1.28, 2.29)	0.96 (0.86, 1.07)	
Small for Gestational Age	Other Maternal IDD	Adequate Prenatal Care	1.90 (1.44, 2.35)	1.90 (1.44, 2.35)	1.00 (1.00, 1.01)	0.3% (-0.7%, 1.7%)
Small for Gestational Age	Other Maternal IDD	Depression/Anxiety	1.92 (1.46, 2.38)	1.92 (1.47, 2.37)	1.00 (0.98, 1.02)	0.1% (-4.3%, 3.7%)
Small for Gestational Age	Other Maternal IDD	Pre-pregnancy BMI	1.96 (1.49, 2.43)	1.96 (1.50, 2.45)	1.00 (0.98, 1.02)	0.3% (-3.8%, 3.2%)
Small for Gestational Age	Other Maternal IDD	Preexisting Hypertension	1.82 (1.40, 2.26)	1.76 (1.36, 2.20)	1.03 (1.01, 1.06)	6.8% (1.8%, 14.7%)
Small for Gestational Age	Other Maternal IDD	Preterm Birth <37 weeks	1.80 (1.36, 2.19)	1.74 (1.32, 2.12)	1.03 (1.00, 1.07)	7.1% (1.2%, 16.0%)
Small for Gestational Age	Other Maternal IDD	Joint	2.23 (1.66, 2.93)	2.08 (1.55, 2.74)	1.07 (1.02, 1.14)	12.6% (4.6%, 23.1%)

Formulas*:

© 2026 Psaras C et al. *JAMA Network Open.*

Direct Effect: The effect on SGA, on the ratio scale, of changing exposure to present vs absent, assuming the mediator the mediator is set to the value it would have taken if the exposure was present. In other words, a direct effect is the effect of exposure, in this case IDD or one of its subtypes, that does not go through the mediator(s) of interest.

Indirect Effect: The effect on SGA, on the ratio scale, of changing the mediator from what it would have been if the exposure was present to what it would have been if the exposure was absent, holding the value of the exposure to present. In other words, an indirect effect is the effect of the exposure that goes through the mediator(s) of interest.

Proportion mediated: (Direct Effect∗(Indirect Effect−1))/(Total Effect−1)

*For further details see *CMAverse* R package documentation.

Joint mediator model for small for gestational age & Any IDD includes: Preterm Birth <37 weeks, Pre-pregnancy BMI, Preexisting Diabetes, Preexisting Hypertension, Depression/Anxiety, Epilepsy, Bipolar/Schizophrenia, Tobacco Use, Adequate Prenatal Care

Joint mediator model for small for gestational age & Maternal Autism includes: Preterm Birth <37 weeks, Pre-pregnancy BMI, Depression/Anxiety, Bipolar/Schizophrenia, Adequate Prenatal Care

Joint mediator model for small for gestational age & Maternal Cerebral Palsy includes: Preterm Birth <37 weeks, Pre-pregnancy BMI, Preexisting Hypertension, Depression/Anxiety, Epilepsy, Bipolar/Schizophrenia, Adequate Prenatal Care

Joint mediator model for small for gestational age & Maternal Chromosomal Difference includes: Preterm Birth <37 weeks, Pre-pregnancy BMI, Preexisting Hypertension, Depression/Anxiety, Adequate Prenatal Care

Joint mediator model for small for gestational age & Maternal Intellectual Disability includes: Preterm Birth <37 weeks, Pre-pregnancy BMI, Preexisting Diabetes, Preexisting Hypertension, Depression/Anxiety, Epilepsy, Bipolar/Schizophrenia, Tobacco Use, Adequate Prenatal Care

Joint mediator model for small for gestational age & Other Maternal IDD includes: Preterm Birth <37 weeks, Pre-pregnancy BMI, Preexisting Hypertension, Depression/Anxiety, Adequate Prenatal Care

*Sample size insufficient for estimates of: Maternal Chromosomal Difference × Bipolar/Schizophrenia; Maternal Chromosomal Difference × Preexisting Diabetes; Maternal Chromosomal Difference × Tobacco Use; Maternal Autism × Epilepsy; Maternal Autism × Preexisting Hypertension; Maternal Autism × Tobacco Use; Maternal Cerebral Palsy × Preexisting Diabetes; Maternal Cerebral Palsy × Tobacco Use; Other Maternal IDD × Bipolar/Schizophrenia; Other Maternal IDD × Epilepsy; Other Maternal IDD × Preexisting Diabetes; Other Maternal IDD × Tobacco Use.

© 2026 Psaras C et al. *JAMA Network Open.*
