# Supplementary material for: Maternal Intellectual and Developmental Disabilities and Infant Outcomes
Source: JAMA Netw Open. 2026 May 27;9(5):e2615005. doi: 10.1001/jamanetworkopen.2026.15005 (PMC13216985; doi:10.1001/jamanetworkopen.2026.15005)
Supplement: Supplement 2. — Data Sharing Statement [file jamanetwopen-e2615005-s002.pdf]

Data Sharing Statement

Psaras. Maternal Intellectual and Developmental Disabilities and Infant Outcomes. *JAMA Netw Open*. Published May 27, 2026. doi:10.1001/jamanetworkopen.2026.15005

Data
Data available: No

Additional Information
Explanation for why data not available: The data that support the findings of this study are available from the California Department of Public Health (CDPH). Restrictions apply to the availability of these data, which were used under license for this study. Authors do not have permission to share data. We direct researchers to the CDPH Center for Health Statistics and Information, and the California Department of Health Care Access and Information for information on requesting and accessing California state data.
